# Physiology governing diatom vs. dinoflagellate bloom and decline in coastal Santa Monica Bay

**DOI:** 10.3389/fmicb.2023.1287326

**Published:** 2023-11-29

**Authors:** Gerid A. Ollison, Sarah K. Hu, Julie V. Hopper, Brittany P. Stewart, Jennifer L. Beatty, David A. Caron

**Affiliations:** ^1^Department of Biological Sciences, University of Southern California, Los Angeles, CA, United States; ^2^Department of Oceanography, Texas A&M University, College Station, TX, United States; ^3^Office of Sustainability, University of Southern California, Los Angeles, CA, United States

**Keywords:** algal bloom, microbial ecology, diatom bloom, dinoflagellate bloom, metatranscriptomics, protistan ecology

## Abstract

Algal blooms on the Southern California coast are typically dominated by diatom and dinoflagellate taxa, and are governed by their physiological responses to environmental cues; however, we lack a predictive understanding of the environmental controls underlying the establishment and persistence of these distinct bloom events. In this study, we examined gene expression among the numerically dominant diatom and dinoflagellate taxa during spring upwelling bloom events to compare the physiological underpinnings of diatom vs. dinoflagellate bloom dynamics. Diatoms, which bloomed following upwelling events, expressed genes related to dissolved inorganic nitrogen utilization, and genes related to the catabolism of chitin that may have prolonged their bloom duration following nitrogen depletion. Conversely, dinoflagellates bloomed under depleted inorganic nitrogen conditions, exhibited less variation in transcriptional activity, and expressed few genes associated with dissolved inorganic nutrients during their bloom. Dinoflagellate profiles exhibited evidence of proteolysis and heterotrophy that may have enabled them to bloom to high abundances under depleted inorganic nutrients. Taken together, diatom and dinoflagellate transcriptional profiles illustrated guild-specific physiologies that are tuned to respond to and thrive under distinct environmental “windows of opportunity.”

## 1. Introduction

Unicellular eukaryotes (protists) conduct nearly half of oceanic primary production, and are particularly important in coastal upwelling regimes located on the eastern boundaries of oceans, high-nutrient ecosystems that have been called the “new production factories of the global ocean” (Field et al., 1998; Capone and Hutchins, 2013). Seasonal algal blooms of large protistan taxa in coastal upwelling regimes feed into short food chains that support the world’s most important fisheries, and constitute an important sink of atmospheric CO_2_. However, some species can form harmful algal blooms (HABs) as a result of high biomass accumulations, or produce toxins that are detrimental to ecosystem and human health (Ryther, 1969; Smith et al., 2018). The timing of blooms along the southern California coast is fairly consistent such that blooms are especially pronounced during the spring when wind-driven upwelling of new nutrients occurs during periods of sufficient light and shallow mixing (Checkley and Barth, 2009). However, we still lack a predictive understanding of the specific environmental controls that govern taxonomic composition or bloom magnitude and duration.

A recent study examined the biotic and abiotic controls of bloom dynamics by tracking daily changes in the protistan community and corresponding physicochemical conditions during two contrasting (diatom vs. dinoflagellate) blooms in Santa Monica Bay off the coast of Southern California (Ollison et al., 2022). A diatom bloom dominated by *Thalassiosira* and *Pseudo-nitzschia* formed following wind-driven upwelling and elevated inorganic nutrient concentrations during 2018, whereas two mixotrophic dinoflagellates, *Margalefidinium* and *Akashiwo,* formed a massive bloom under lower inorganic nutrient concentrations in 2019 (Ollison et al., 2022). Network analysis and direct observations via microscopy revealed that parasites were actively infecting diatom taxa in 2019 (Ollison et al., 2022). The authors speculated that parasite attack on diatoms during 2019, but not in 2018, may have suppressed diatoms or produced alternative sources of nutrients that influenced the subsequent dinoflagellate bloom success.

Algal bloom dynamics are governed by physiological responses to environmental cues and many studies have illustrated that diatoms and dinoflagellates have distinct ecological niches (Irwin et al., 2012). Diatoms are thought to thrive under cool nutrient-rich conditions as a result of their size and high capacity for utilizing dissolved inorganic nitrogen and thus typically dominate under nutrient upwelling conditions during the spring (Margalef, 1978; Falkowski et al., 2004; Armbrust, 2009). Conversely, dinoflagellate blooms typically occur under warm, nutrient poor, stratified conditions (Margalef, 1978; Smayda and Reynolds, 2001). Additionally, many dinoflagellate taxa such as *Margalefidinium* and *Akashiwo* are mixotrophs, having the ability to consume prey in addition to conducting photosynthesis to support their nutritional demands (Smayda, 2010; Caron, 2016; López-Cortés et al., 2019; Yang et al., 2020). However, relatively few studies have examined the physiological processes that govern natural blooms of these algal groups at the level of gene transcription (Alexander et al., 2015b; Wurch et al., 2019; Zhang et al., 2019; Metegnier et al., 2020). For example, Zhang et al. (2019) examined genes associated with the regime shift from diatom to dinoflagellate dominance and identified key nutritional differences. Additionally, Wurch et al. (2019) examined an *Aureococcus* bloom and highlighted the significance of phosphorus in controlling its magnitude and duration.

Metatranscriptomic sequencing has become a tractable approach for examining the physiological activity of whole protistan communities *in situ*, and is largely a result of technological, computational, and database advances. For example, the Marine Microbial Eukaryotic Transcriptome Sequencing Project (MMETSP) has produced over 650 assembled, annotated, and publically available transcriptomes of non-model protists (Keeling et al., 2014). Metatranscriptomic investigations of natural communities have begun to improve our understanding of physiological adaptations that underpin species responses to diverse environmental stimuli (Wurch et al., 2014; Geisen et al., 2015; Alexander et al., 2015a,b; Cohen, 2017; Hu et al., 2018; Zhang et al., 2019; Harke et al., 2021; Cohen et al., 2022). For example, Wurch et al. (2019) examined the physiological underpinnings of an *Aureococcus* bloom and reported the unexpected importance of phosphorus in controlling bloom dynamics (Wurch et al., 2019).

This current study extends findings from Ollison et al. (2022) by examining the gene expression patterns associated with temporal changes in relative abundances of dominant diatom and dinoflagellate taxa. Ordination of diatom and dinoflagellate transcriptomes revealed guild-specific physiologies, while gene co-expression network analysis and differential expression analysis illustrated periodic shifts in physiological priorities that may have influenced bloom persistence under nutrient deficiency. Our analyses indicate that while diatoms responded to inorganic nutrient enrichment from upwelling, and exhibited alternative strategies for inorganic nutrient acquisition that may have prolonged bloom duration following nutrient drawdown, dinoflagellates gene expression indicated that they may exploit organic sources of nutrients or prey (i.e., mixotrophy) to reach bloom proportions under depleted inorganic nutrient conditions.

## 2. Methods

The physiological underpinnings of two distinct algal blooms in waters off the coast of southern California — a diatom-dominated bloom in spring 2018 and a dinoflagellate-dominated bloom during spring 2019 — were examined using eukaryotic metatranscriptomic datasets. Gene expression of dominant diatom and dinoflagellate taxa were filtered and examined from whole community gene expression during four periods of growth: pre-bloom; a period of rapid increases in chlorophyll concentration and relative abundance; a period of sustained abundances (determined from chlorophyll concentrations and maximum relative abundances); and bloom decline. The dominant diatom and dinoflagellate taxa during each year were identified previously via light microscopy and 18S rRNA gene transcript sequencing (Ollison et al., 2022). The diatom genera *Thalassiosira* and *Pseudo-nitzschia* were the numerically dominant taxa during 2018, whereas two gymnodiniacean dinoflagellate genera, *Margalefidinium* and *Akashiwo* were dominant during 2019. In the present study, gene expression from these dominant taxa were examined on five days during each year that spanned the aforementioned bloom periods. Sequences of these dominant taxa were filtered from whole community gene expression, assigned KEGG functional annotations, and examined using multi-variate, differential expression, and weighted gene co-expression network analyses to compare the physiological processes that governed shifts in biomass during each year.

### 2.1. Sample collection

Protistan communities and physicochemical parameters in Santa Monica Bay, California, were sampled daily throughout blooms dominated by diatoms and dinoflagellates during spring 2018 and 2019, respectively (Ollison et al., 2022). Briefly, sampling from the Santa Monica Pier (SMP) was conducted daily, April 16th through the 30th in 2018 (15 consecutive days), and April 13th through May 6th in 2019 (22 days; no sample was collected on the 14th April 2019). The taxonomic composition of metabolically active protists was assessed during each bloom using 18S rRNA transcript sequencing and light microscopy, chlorophyll *a* assessed from whole seawater (chlorophyll) via fluorometry of acetone extracted chlorophyll, and nitrate + nitrite and phosphate concentrations were measured from 0.2 μm filtered seawater via flow injection (see Ollison et al., 2022). Sampling periods are hereafter referred to as 2018 and 2019, and are synonymous with diatom and dinoflagellate dominated blooms, respectively.

Temporal changes in taxonomic abundances and physicochemical parameters discussed in Ollison et al. (2022) informed our metatranscriptomic sequencing approach to assess the four distinct bloom phases from each year: (1) Pre-bloom, (2) onset of increasing relative abundances (based on 18S-V4 rRNA gene transcript sequencing, see below) and chlorophyll concentration, (3) sustained relative abundances (maximum relative abundances and chlorophyll concentration), (4) and bloom decline (decline in relative abundances and chlorophyll concentration).

### 2.2. Sample processing

Eukaryotic metatranscriptomic community analyses on each sampling date were performed in triplicate on 2 L seawater samples, which were pre-filtered through Nitex mesh (80 μm) to exclude most metazoa, and collected onto 47 mm GF/F glass fiber filters (nominal pore size 0.7 μm; Whatman, International Ltd. Florham Park, NJ) to capture the unicellular eukaryote community while excluding most metazoa. The filters were placed in 15 mL RNAse-free Falcon tubes containing 1.5 mL of RLT buffer (Qiagen, #79216) + betamercaptoethanol (ThermoFisher, #21985023), immediately flash frozen in liquid nitrogen, and subsequently stored at −80°C until RNA extraction.

### 2.3. RNA extraction and sequencing

Total RNA was extracted per a previously established protocol (Ollison et al., 2021). Briefly, each GF/F filter was shredded by vortexing after the addition of silica beads to each tube containing a GF/F filter and lysis buffer. The mixture was transferred to a syringe that was used to obtain the lysate from the filter/water slurry. RNA was extracted from the lysate via Qiagen All Prep DNA/RNA Mini Kit (Qiagen, #80204) per manufacturer instructions. Genomic DNA was removed prior to RNA extraction using an RNase-Free Qiagen DNase (Qiagen, #79254). RNA was reverse transcribed to cDNA using the Bio-Rad iScript Reverse Transcription Supermix with random hexamers (Bio-RAD, #170–8,840).

Sequence library preparation was performed at the University of Southern California’s UPC Genome Core facility using Kapas Stranded mRNA library preparation kit with poly-A tail selection beads to concentrate eukaryotic mRNA (Kapa Biosystems, Inc., Wilmington, MA #KK8420). RNA libraries were quality checked (Agilent bioanalyzer 2,100) prior to sequencing. Sequencing was conducted on four NextSeq High Output PE 150 runs.

### 2.4. Sequence processing

Sequence adapters, low quality bases (phred score below 10 within a 25 bp sliding window), and sequences shorter than 50 bp were removed using Trimmomatic v.0.32 (Bolger et al., 2014). rRNA and mRNA were sorted from quality-filtered reads using SortMeRNA (v2.1) (Kopylova et al., 2012). Taxonomic classification was subsequently assigned to sorted rRNA reads using the Protist Ribosomal Reference (v.11) database via uclust at 97% identity (Guillou et al., 2013).

Messenger RNA sequences from combined replicate samples were co-assembled to contiguous sequences (contigs) using MEGAHIT v. 1.0.3 (Li et al., 2015) with default parameters. Contigs were assigned taxonomic identities and gene function IDs (Kyoto Encyclopedia of Genes and Genomes (KEGG) orthology IDs) using diamond BLASTX on sensitive mode using an e-value cutoff of 0.001 to a customized cDNA reference database that augmented the Marine Microbial Eukaryote Transcriptome Sequencing Project (MMETSP) database with other publicly available genomes and transcriptomes (EukZoo) (Keeling et al., 2014; Buchfink et al., 2015). Hits with bit scores within the top 95% were assigned taxonomic and functional annotations, and only transcripts with functional annotations were used in downstream analysis of physiological activity. More information about the EukZoo is available with download at the Zenodo repository: http://doi.org/10.5281/zenodo.1476236. Transcript abundances were quantified using Salmon v. 0.11.3 (kmer size = 31) (Patro et al., 2017).

### 2.5. Sequence analysis

Genes expressed by numerically dominant diatom and dinoflagellate taxa—i.e. those with the greatest relative abundances—were examined to assess shifts in physiological activity during their respective bloom. Relative abundances of taxa were characterized in this study via metatranscriptome-derived 18S rRNA gene transcripts and previously via 18S-V4 gene transcript sequencing-based characterization from Ollison et al. (2022). Genes expressed by the numerically dominant diatom taxa during 2018 were bioinformatically isolated from total community gene expression during both years. Genes expressed by the numerically dominant dinoflagellates during 2019 were isolated from whole community gene expression during both years. Two samples—one technical replicate from Day 1 and Day 12 of 2019—were excluded from further analysis due to low read abundances after filtration for gymnodiniacean dinoflagellates. Samples from 2018 to 2019 were normalized separately due to differences in sequenced reads (2019 had more), and each diatom and dinoflagellate taxon was normalized independently prior to differential expression analysis using the trimmed mean of m-value (TMM) method via edgeR v3.30.3 (Robinson et al., 2010) in order to account for changes in relative proportions during the bloom.

Differential expression analysis was conducted by pairing algorithms for calculating differential expression via edgeR (Robinson et al., 2010) and DESeq2 (Love et al., 2014), where an estimate of common dispersion of normalized counts (edgeR) and raw counts (DESeq2) was calculated across the three technical replicates. Only transcripts that met three criteria were considered differentially expressed and retained for downstream analysis: one, determined to be significantly differentially expressed by both algorithms (adjusted *p*-values <0.01); two, differentially expressed in the same direction (positive vs. negative log_2_ fold change) in both algorithms; three, the difference in log fold change predicted by both algorithms less than |1|. This last arbitrary cutoff was included to minimize inclusion of differential expression predictions with low-precision and thereby improve the accuracy of interpretation of average log_2_ fold changes.

Weighted gene co-expression networks were also constructed to examine the association of expression modules, which are clusters of genes with similar expression patterns, with bloom periods and their correlation with environmental parameters during both blooms using the WGCNA package (Langfelder and Horvath, 2008). Co-expression networks were constructed using powers of 7 and 8 for diatom and dinoflagellate network construction, respectively. Genes with similar expression patterns were grouped into modules using the *cutreeDynamic* function with *deepsplit*, *pamRespectsDendro*, and *minClusterSize* flags set to 2, FALSE, and 10, respectively. The *cutheight* flag was left at the default parameter. Modules were clustered (average method) using Pearson correlations of module eigenvalues (first principal component of the expression matrix), and highly similar modules were manually merged for subsequent analyses. Functional enrichment of KEGG terms within WGCNA modules was conducted using ClusterProfiler, which queries current KEGG databases for functional enrichment annotation (Wu et al., 2021). Functional enrichment terms were manually curated to minimize redundancy and improve interpretation. Additionally, enrichment terms associated with human diseases were aggregated as “Human Disease Pathways.”

Ordination was executed using the vegan package, v.2.7 (Dixon, 2003). All artwork was visualized in R studio, and most data wrangling and visualization was executed using tools from the Tidyverse (Wickham et al., 2019). All custom scripts used in data analysis are available at: https://github.com/theOlligist/SMP-metaT-Pairing-edgeR-and-DESeq-differential-expression.

## 3. Results

### 3.1. Protistan community composition and dynamics

The protistan community was sampled daily following two upwelling events that resulted in major phytoplankton blooms (both >12 μg/L chlorophyll), where diatoms were dominant during the spring of 2018 and dinoflagellates during the spring of 2019 (Ollison et al., 2022). 18S rRNA transcript sequencing and light microscopy illustrated that *Thalassiosira* and *Pseudo-nitzschia* were the numerically dominant taxa during 2018 and two gymnodiniacean dinoflagellates, *Margalefidinium* and *Akashiwo,* dominated during 2019 (Ollison et al., 2022). During each bloom, whole community gene expression was examined during four distinct periods (see methods). An average of 27.2 million quality filtered reads per sample produced an average of 2.5 million co-assembled contiguous sequences per sampling day, where 50% of sequence reads were assigned taxonomy and 30% were assigned KEGG orthology annotations (Supplementary Table S1).

**Figure 1:**
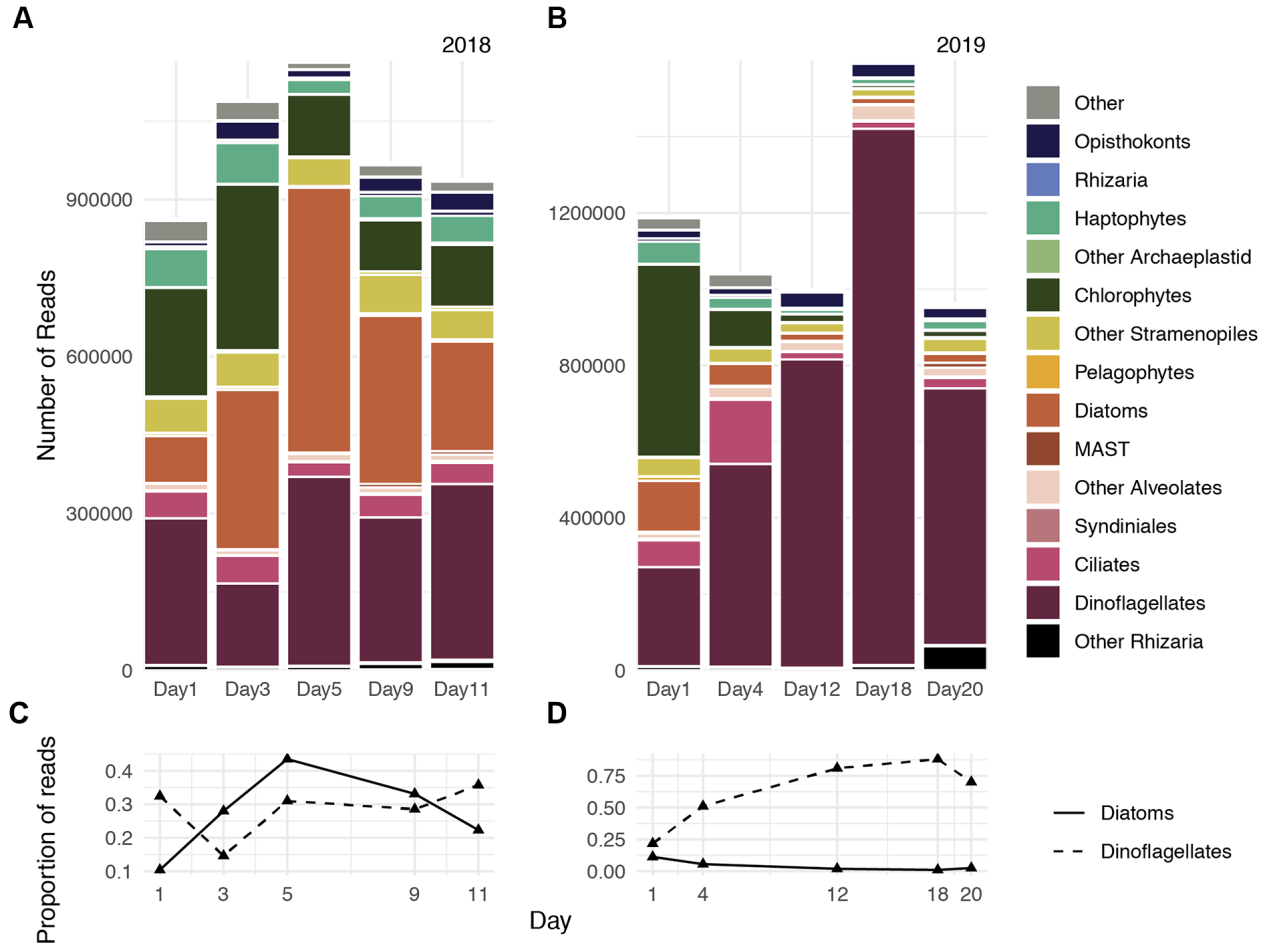
Gene expression dynamics of protistan communities at the Santa Monica Pier on five days during spring 2018 **(A)** and spring 2019 **(B)**. The proportion of genes transcribed by the diatom assemblage (solid line) and dinoflagellate assemblage (dashed line) during 2018 **(C)** and 2019 **(D)**.

Consistent with dynamics characterized by 18S-V4 gene transcripts reported by Ollison et al. (2022), Metatranscriptome-derived rRNA illustrated that during 2018, diatoms accounted for approximately 10% of the protistan community on Day 1, which increased to 25% on Day 3, and reached maximum relative abundances on Day 5 (45%), followed by a gradual declined through Day 9 (30%) and Day 11 (20%, Figures 1A,C). Chlorophytes and haptophytes accounted for approximately 30% of the community during Day 1 and reduced to approximately 15% on Day 9 and Day 11. Dinoflagellates comprised approximately 25% of the community on average and accounted for slightly more of the community during advanced bloom stages during the 2018 study period (Figures 1A,C).

For most of the 2019 sampling period, dinoflagellates accounted for greater than 50% of the protistan community. Dinoflagellates accounted for approximately 20% on Day 1, rapidly increased in relative abundances through Day 4 (50%) and Day 12 (75%), reached peak relative abundances (85%) on Day 18, and declined to 70% of the community on Day 20 (Figures 1B,D). Transcripts assigned to other photosynthetic taxa (chlorophytes, diatoms, haptophytes) greatly declined in relative abundance after Day 1, and collectively constituted approximately 10% of the protistan community throughout the remainder of sampling period during 2019. Changes in chlorophyll concentrations reported by Ollison et al. (2022) recapitulated increases, maxima, and declines of both diatom and dinoflagellate relative abundances during 2018 and 2019, respectively (Figure 2 of Ollison et al., 2022).

**Figure 2:**
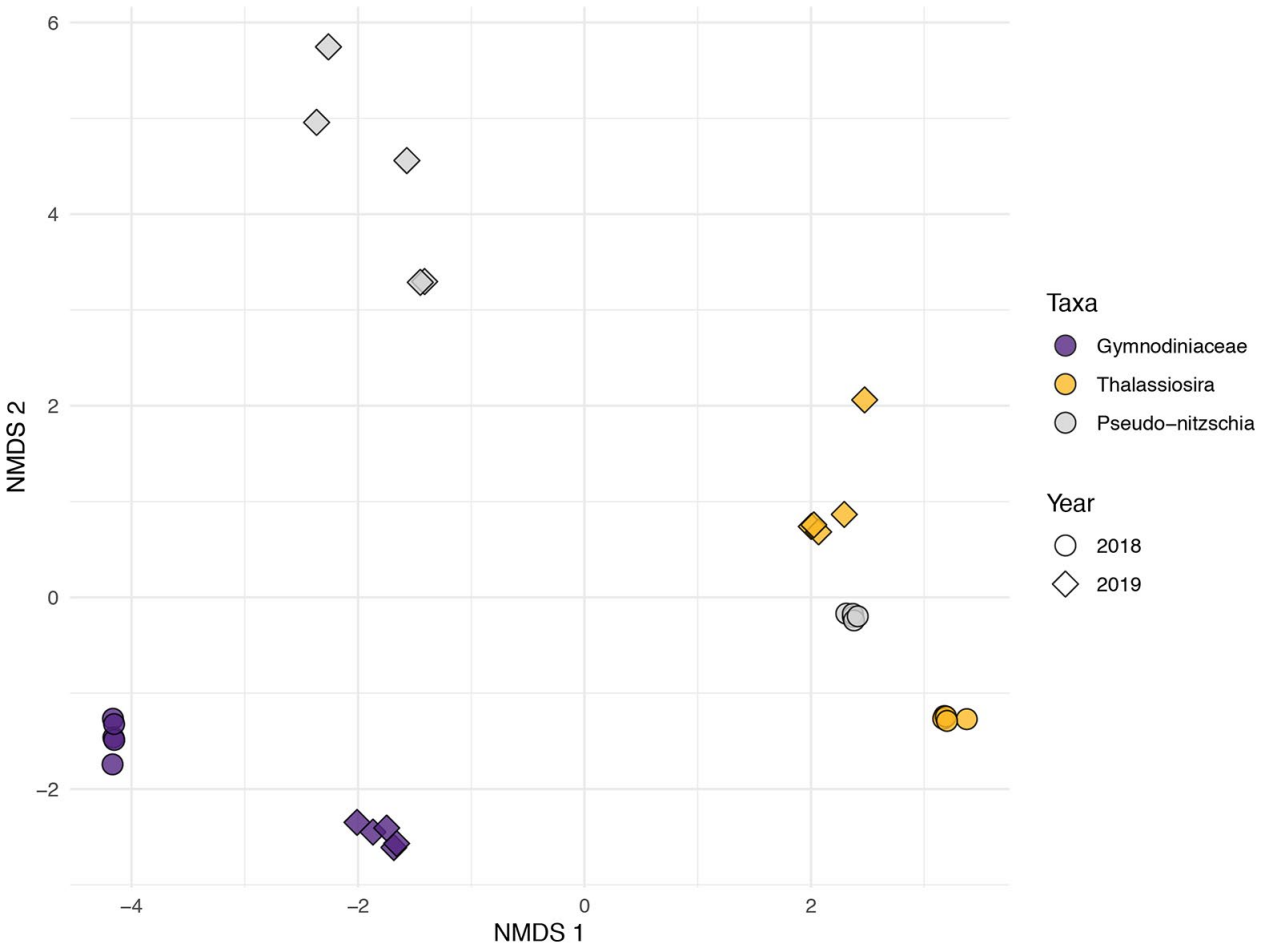
Non-metric multidimensional scaling (Bray-Curtis) of gymnodiniacean dinoflagellate (Gymnodiniaceae; purple) and diatom (*Thalassiosira* in yellow and *Pseudo-nitzschia* in grey) KEGG ID’d expression profiles during 2018 (circles) and 2019 (diamonds).

Accordingly, mostly diatom reads were recovered during 2018, whereas mostly dinoflagellate reads were recovered during 2019 (Figures 1C,D). Approximately 60% of diatom reads were classifiable during 2018, and *Thalassiosira* and *Pseudo-nitzschia* constituted the largest fraction of classifiable diatom transcripts. Both taxa also accounted for a substantial fraction of diatom reads during 2019, although diatom reads cumulatively accounted for only 4% of the total community during that year compared with 28% during 2018. Approximately 30% of dinoflagellate reads were classifiable during 2019, and Gymnodiniaceae, the dinoflagellate family that encompasses both *Margalefidinium* spp. and *Akashiwo* spp., accounted for approximately 30% of classifiable dinoflagellate reads on average during both years. Hereafter we refer to the dominant dinoflagellates examined in this study (*Margalefidinium* and *Akashiwo*) as gymnodiniacean dinoflagellates.

### 3.2. Contrasting diatom and dinoflagellate physiological profiles

Gene expression of *Thalassiosira*, *Pseudo-nitzschia*, and gymnodiniacean dinoflagellates was examined during aforementioned growth phases of their respective bloom and non-bloom conditions to contrast the physiological underpinnings of diatom vs. dinoflagellate growth dynamics associated with algal blooms in the two years.

The functional composition of diatom and dinoflagellate expression profiles revealed that approximately 2.2 million diatom transcripts corresponded to 22,231 KEGG orthology (KO) IDs during their bloom year (2018), and approximately 3.4 million diatom transcripts corresponded to 12,573 KO IDs during 2019. Gymnodiniacean dinoflagellates accounted for approximately 226,000 transcripts that corresponded to 2,402 KO IDs during their bloom year (2019) and approximately 33,200 transcripts that corresponded to 2,388 KO IDs during 2018.

Non-metric multidimensional scaling (Bray-Curtis) of five daily samples during the two years for the three algal taxa (two diatom genera and one dinoflagellate family) based on their composition of KO IDs (30 total functional profiles) produced three broad groupings (Figure 2). There was a clear separation between the functional profiles of dinoflagellates (Figure 2, purple symbols) and diatoms. Additionally, *Thalassiosira* from both years grouped with *Pseudo-nitzschia* profiles obtained during 2018, while *Pseudo-nitzschia* during 2019 formed one distinct, loosely clustered grouping (Figure 2, gray diamonds). Samples for all three taxa formed secondary groupings according to year, where *Pseudo-nitzschia* profiles exhibited the greatest dissimilarity between years.

**Figure 3:**
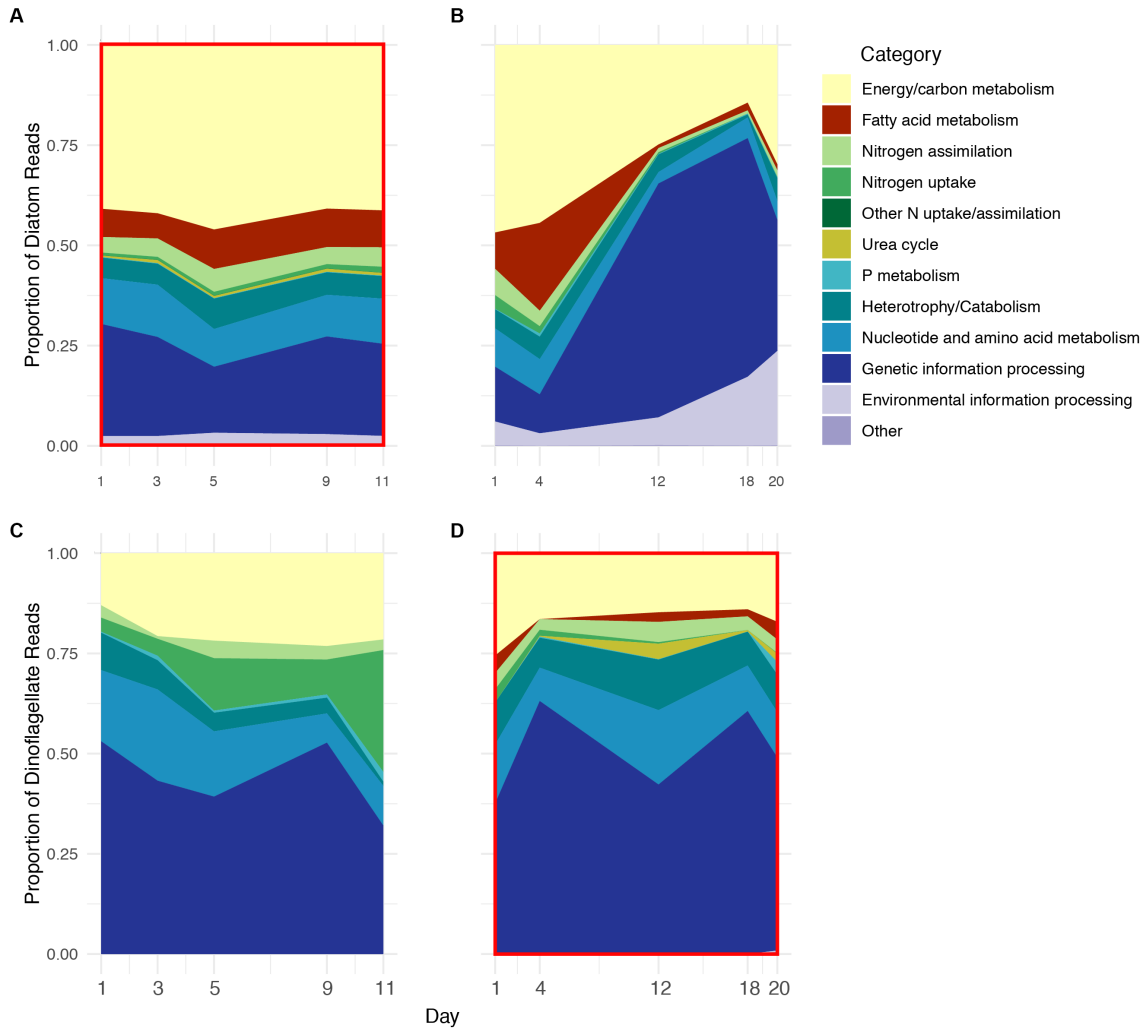
Relative proportion of KEGG ID’d transcripts on five days during 2018 **(A,C)** and 2019 **(B,D)** grouped as functional categories that were expressed by diatoms **(A,B)** and dinoflagellates **(C,D)** during 2018 and 2019. Red boxes indicate years of numerical dominance of diatoms **(A)** and dinoflagellates **(D)**.

KO IDs were assigned to functional category groupings, and the functional profiles (based on these groupings) of *Thalassiosira* and *Pseudo-nitzschia* were combined to further compare diatom vs. dinoflagellate physiology. Diatom functional profiles from 2018 to 2019 contained the same functional categories although proportions varied between years and somewhat between sampling days, particularly during 2019 (Figures 3A,B). During 2018, the proportions of functional categories were relatively stable across all stages, but during 2019 they exhibited a pronounced shift in proportions after Day 4. The shift was mostly due to the sharp increase in transcripts associated with the genetic information processing category, which increased in proportion from ~10% on Day 1 to ~60% on Day 12 during 2019. Approximately 90 and 98% of transcripts associated with this category coded for ribosomal proteins in *Thalassiosira* and *Pseudo-nitzschia*, respectively (Figures 3A vs. B; Supplementary Table S1). Conversely, during 2018 the genetic information processing category consistently accounted for approximately 25% of diatom profiles. Transcripts associated with energy and carbon metabolism (photosynthesis, Calvin cycle, glycolysis, TCA cycle, and fatty acid metabolism) accounted for approximately half of diatom expression profiles during 2018 and early stages of 2019, but only accounted for approximately 25% during advanced stages of 2019 (Figures 3A,B; Supplementary Figures S1A,B).

Transcripts associated with inorganic nitrogen uptake and assimilation accounted for approximately 5% diatom profiles on average, where the proportion of transcripts associated with nitrogen assimilation (GS/GOGAT) was greater than those associated with dissolved inorganic nitrogen uptake (AMT and NRT) during both years (Supplementary Figures S2A,B). The overall proportion of transcripts associated with nitrogen assimilation was stable across 2018, but decreased from approximately 8% on Day 1 to less than 1% after Day 4 during 2019 (Figures 3A,B). In contrast to inorganic nitrogen, transcripts associated with phosphate and the urea cycle accounted for less than 1% of the diatom profiles during both years.

Transcripts associated with catabolic processes accounted for approximately 5% of diatom profiles throughout 2018 and the majority of 2019, and lysosomal processes accounted for most of this category (Figures 3A,B; Supplementary Figures S1A,B). Transcripts associated with the catabolism of chitin, which is a nitrogen-rich polymer produced by diatoms and diverse eukaryotic lineages, and endocytosis also accounted for approximately 1% of the diatom profiles on average during both years, a feature that was unique to *Thalassiosira* (Supplementary Figure S3).

Transcripts associated with nucleotide and amino acid metabolism, and environmental information processing categories collectively accounted for approximately 10% of diatom profiles throughout 2018 and prior to Day 12 of 2019. The majority nucleotide and amino acid metabolism-associated transcripts were represented by nucleoside-diphosphate kinase and aspartate kinase, which accounted for 32 and 13% of this category, respectively, on average (Figures 3A,B).

Dinoflagellate profiles observed during 2018 and 2019 also varied in proportion of categories as well as composition (i.e., presence/ absence), where dinoflagellate profiles during 2018 contained fewer categories than 2019 (Figures 3C,D). Transcripts associated with the genetic information processing category accounted for the majority of dinoflagellate profiles (~50%) during both years with relatively low variability compared to diatoms during 2019. The majority of transcripts in this category coded for snRNPs (~23%) and ribosomal proteins (~71%).

The proportion of transcripts associated with energy and carbon metabolism accounted for ~20% of dinoflagellate profiles during both years, less than half of the average proportion observed in diatoms (Figures 3C,D). Notably, dinoflagellate profiles during 2019 contained several additional energy and carbon metabolism-related categories that were not present during 2018. For example, transcripts associated with fatty acid metabolism were unique to dinoflagellate profiles during 2019, with the largest proportions observed following Day 4, during the latter stages of the bloom (Supplementary Figure S3D).

Transcripts associated with dissolved nitrogen uptake were highly variable between years in dinoflagellate profiles. During 2018, transcripts associated with nitrogen uptake (NRT) increased from 3% on Day 1 to greater than 25% by Day 11; however, during 2019, transcripts associated with nitrogen uptake decreased to less than 1% of dinoflagellate profiles after Day 1 (Figures 3C,D; Supplementary Figures S1C,D). Interestingly, transcripts associated with nitrogen assimilation (GS/GOGAT) remained approximately 5% of dinoflagellate profiles during both years. There were no transcripts associated with the urea cycle during 2018, but during 2019 they accounted for approximately 1% of dinoflagellate profiles on average, with maximum expression (~3%) on Day 12. The majority of urea cycle transcripts were for arginase, a nitrogen-rich intermediate for ornithine and urea biosynthesis. Similar to diatoms, transcripts associated with phosphate were a minor component of dinoflagellate profiles during both years, with maximum transcription (~1%) during 2019 on Day 20.

Transcripts associated with catabolism and heterotrophy accounted for ~6% of dinoflagellate profiles on Day 1 and decreased to less than 1% by Day 11 during 2018; the majority of transcripts in this category during 2018 were associated with SNARE complex and phagosome maturation (Figures 3C,D). Conversely, throughout 2019, catabolism and heterotrophy accounted for ~10% of dinoflagellate profiles, and the majority transcripts in this category were associated with lysosomal processing and phagosome maturation (Figure 3D, Supplementary Figure S1D).

The proportion of transcripts associated with nucleotide/amino acid metabolism accounted for approximately 12% of dinoflagellate profiles on average during both 2018 and 2019, and the majority of these transcripts were associated with GDP-D-mannose (~50%), and uroporphyrinogen decarboxylase (~11%).

### 3.3. Patterns of differential gene expression during diatom and dinoflagellate blooms

Analysis of genes that exhibited significant differential expression by diatoms and dinoflagellates was conducted using Day 1 from each sampling period as the pre-bloom baseline from which to contrast gene expression levels during subsequent growth phases of their respective blooms (diatoms in 2018 and dinoflagellates in 2019). Examination of genes that exhibited significant differential expression (DEGs) by diatoms and dinoflagellates revealed that diatoms exhibited 1,008 total DEGs where approximately 60% exhibited negative log_2_ fold changes relative to baseline gene expression (under-expressed; Supplementary Figure S4), and dinoflagellates exhibited only 225 DEGs with the majority exhibited positive log_2_ fold changes relative to baseline gene expression (over-expressed; ~72%; Supplementary Figure S5, Supplementary Table S2).

The number of DEGs was unequally distributed across either study period for both taxa. For diatoms, bloom development (Day 3) exhibited the fewest proportion of DEGs (~5%) whereas Day 5, which coincided with maximum chlorophyll concentrations, accounted for approximately 50% of total DEGs; most were under-expressed (Supplementary Figures S4A,B). Dinoflagellates also expressed the majority of DEGs during the period of maximum chlorophyll concentrations on Day 18 of 2019(~30%), although most were over-expressed, and the early phase of bloom establishment (Day 4) also accounted for the fewest total DEGs as was also the case for diatoms (~18%; Supplementary Figures S5A,B).

**Figure 4:**
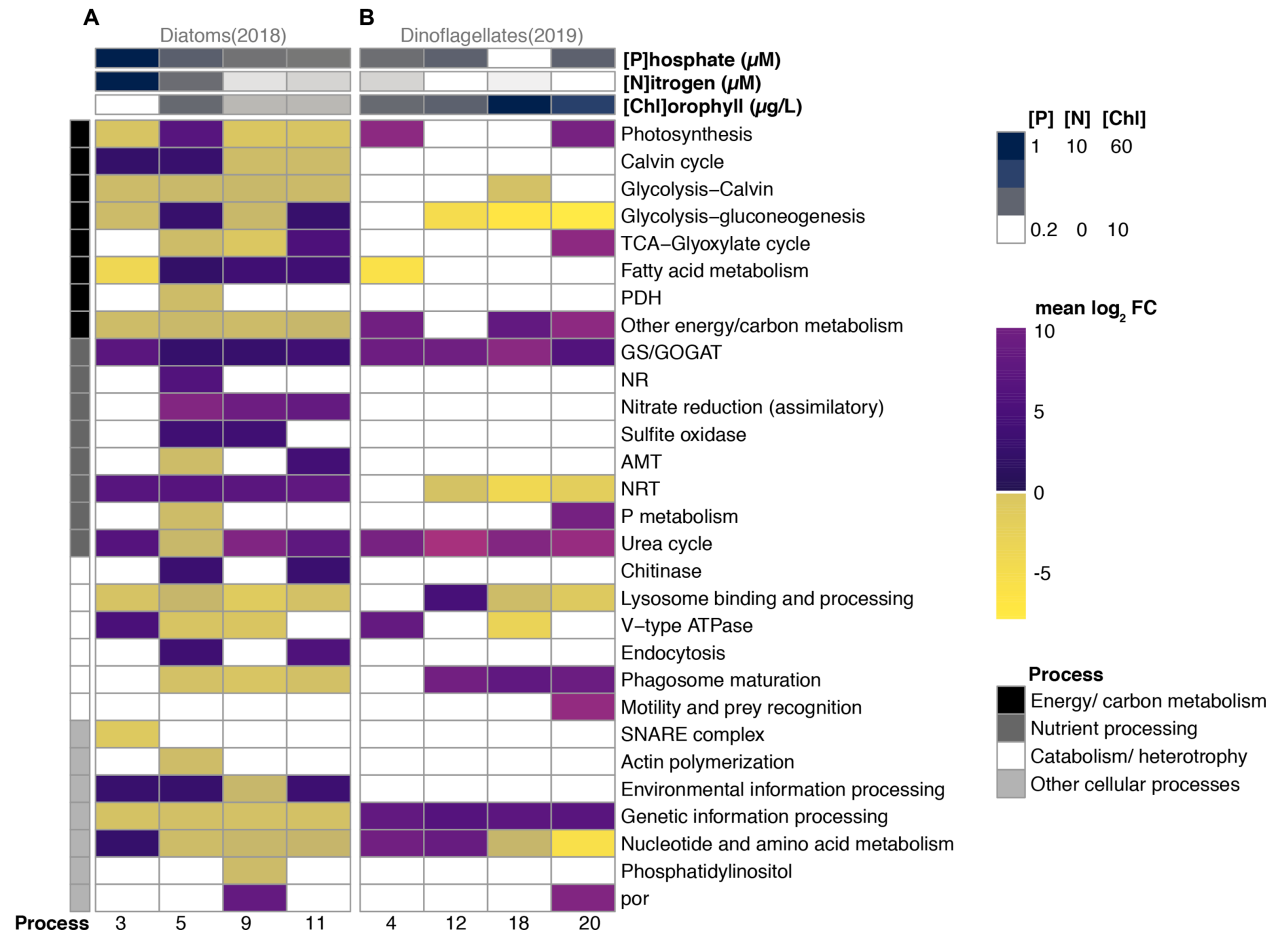
Differential expression (mean log_2_ fold change relative to Day 1) of 36 functional categories grouped into four broad physiological processes of numerically dominant diatoms **(A)** vs. dinoflagellates **(B)** during periods (*x*-axis) of their bloom in 2018 vs. 2019, respectively. Nitrate + nitrite (Nitrogen), phosphate, and chlorophyll concentrations are shown in the top three rows. The four physiological processes are color-coded in the left-hand vertical column.

Diatom DEGs corresponded to 28 KO categories across four broad scale physiological groupings, and the average log_2_ fold change of most categories exhibited shifts in direction (positive vs. negative) and magnitude across bloom stages (Figure 4A). Most genes exhibiting differential expression in diatoms were associated with energy/carbon metabolism, a category that also accounted for the majority of diatom transcription profiles (Figure 3A). Whereas enzymes functioning in both glycolysis and the Calvin cycle (glycolysis-Calvin), and other metabolism-related transcripts lacking further annotation remained under-expressed throughout the 2018 sampling period, the majority of the other energy/carbon metabolism-related categories changed magnitude and direction across bloom phases relative to Day 1 (Figure 4A). Generally, DEGs in this category were under-expressed during 2018; however, fatty acid metabolism was over-expressed for the majority of 2018, and the most over-expression of various energy/carbon metabolism categories was observed during Day 5, the period of maximum chlorophyll (number of black processes in Figure 4A).

During Day 3, which contained the fewest diatom DEGs, transcripts associated with the TCA cycle maintained constitutive expression, and the majority of DEGs related to energy/carbon metabolism were under-expressed (i.e., the average log2 fold change of each DEG category), where DEGs associated with the Calvin cycle were the exception, and the fatty acid metabolism category exhibited the strongest under-expression (Figure 4A). During Day 5, which coincided with maximum relative abundances of transcripts, all metabolism-related categories exhibited differential expression of which half were over-expressed. Notably, DEGs associated with photosynthesis and glycolysis DEG categories were over-expressed relative to Day 1, along with fatty acid metabolism which also exhibited increasing log_2_ fold changes during subsequent periods. DEGs associated with primary production were subsequently under-expressed through Day 9 and Day 11, a period when only fatty acid metabolism (Day 9) and/or cellular respiration (glycolysis and TCA cycles; Day 11) was over-expressed.

Relatively few categories related to nutrient processing (3 of 8 categories) and catabolism/heterotrophy (2 of 6 categories) were differentially expressed on Day 3, the period that coincided with rapid increases in diatom relative abundances (Figures 1A, 4A). All three nutrient processing categories exhibiting differential expression (GS/GOGAT, NRT, and Urea cycle) were over-expressed and contained categories associated with both dissolved inorganic nitrogen uptake (NRT), assimilation (GS/GOGAT), and dissolved organic nitrogen utilization (urea cycle; Figure 4A). NRT, GS/GOGAT and DEGs associated with assimilatory nitrate reduction (ferredoxin-nitrite reductase) maintained over-expression throughout the majority of 2018, although categories related to nitrogen assimilation decreased in magnitude of differential expression with time (Figure 4A). The urea cycle category was over-expressed throughout the 2018 sampling period by diatoms with the exception of Day 5.

During Day 5, all catabolism and nutrient-related categories expressed by diatoms were differentially expressed, and nearly half were under-expressed in both groupings, a pattern similar to the energy/carbon metabolism grouping. Notably categories associated with nitrate assimilation (GS/GOGAT, NR, assimilatory nitrate reduction (ferredoxin-nitrite reductase), and sulfite oxidase) were over-expressed, whereas only one category associated with nitrogen uptake (NRT) was over-expressed; AMT, urea cycle, and P metabolism were all under-expressed on Day 5 (Figure 4A). Additionally, whereas most DEGs associated with catabolism and heterotrophy remained under-expressed throughout 2018, chitinase and endocytosis were only over-expressed when found significantly differentially expressed on Day 5 and Day 11. Most categories associated with other cellular processes were under-expressed after Day 3.

Dinoflagellates during 2019 exhibited differential expression (relative to Day 1) across 17 categories, a number smaller than what was observed in diatoms (28 categories; Figure 4B). Six categories associated with energy/carbon metabolism exhibited differential expression during 2019. On Day 4, photosynthesis, fatty acid metabolism, and DEGs associated with energy/carbon metabolism but lacking further functional classification (“Other energy/carbon metabolism” category) were the only categories exhibiting differential expression. Transcripts associated with fatty acid metabolism were only under-expressed on average on Day 4 (2019), but those associated with glycolysis-gluconeogenesis were under-expressed for the majority of the sampling period. Conversely, transcripts associated with photosynthesis and other carb/energy metabolism were over-expressed on average during Day 4 and during other advanced bloom phases (Figure 4B).

Four functional categories associated with nutrient processing exhibited differential expression. Whereas GS/GOGAT and urea cycle were over-expressed throughout the 2019 sampling period after Day 1, NRT were under-expressed after Day 4. DEGs associated phosphate metabolism (P metabolism) were over-expressed on Day 20, a period of bloom decline during 2019 (Figure 4B).

The majority of differentially expressed categories associated with catabolism/heterotrophy were over-expressed in dinoflagellates relative to Day 1 expression levels (Figure 4B). V-type ATPase, which acidifies lysosomes, was over-expressed during periods of increased relative abundances, but was subsequently under-expressed on Day 18, a day that coincided with maximum chlorophyll concentrations during 2019. Lysosomal processing was over-expressed on Day 12 and subsequently under-expressed throughout Day 18 and Day 20. Phagosome maturation DEGs were over-expressed following Day 4, and DEGs associated with motility were over-expressed on Day 20.

The majority of categories associated with other cellular processes were over-expressed; however, DEGs associated with nucleotide and amino acid metabolism were over-expressed during periods of increasing relative abundances (Day 2 and Day 12) and under-expressed during periods coincident with sustained growth (Day 18) and decline (Day 20). Notably, por was also over-expressed at the onset of bloom decline (Day 20), a pattern similar to observations in diatoms during 2018 (Figure 4).

### 3.4. Contrasting diatom and dinoflagellate gene expression modules

Weighted gene co-expression network analysis (WGCNA) was conducted using diatom (*Thalassiosira* and *Pseudo-nitzschia*) and gymnodiniacean dinoflagellate gene expression to further investigate patterns of gene expression that were unique to each growth period and to identify correlations with environmental parameters. WGCNA is a technique for identifying clusters of genes with similar expression dynamics (co-expression modules) and correlating the module eigengenes, which represent the first principal component of the expression matrix, with environmental parameters (Langfelder and Horvath, 2008).

Approximately 26,000 diatom KEGG IDs during 2018 (15,781, *Thalassiosira*; 10,278 *Pseudo-nitzschia*) and 2,400 gymnodiniacean dinoflagellate KEGG IDs during 2019 were clustered into 31 and 22 modules, respectively, of varying sizes (Supplementary Figures S6A, S7A). Nine and five diatom and dinoflagellate modules, respectively, exhibited strong correlations with sampling periods that were consistent across all replicates and were kept for further analysis (Supplementary Figures S6B, S7B).

**Figure 5:**
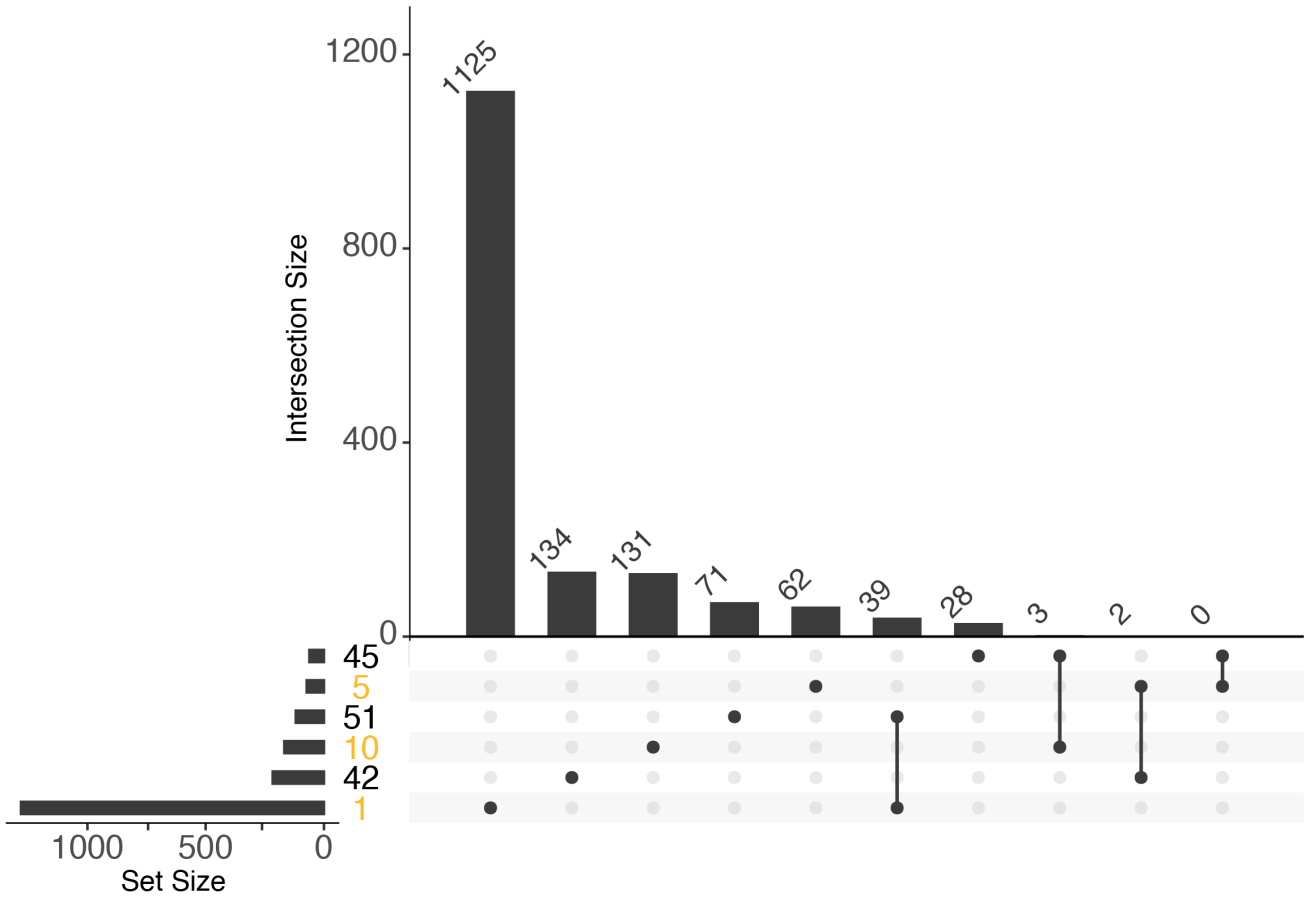
UpSet plot illustrating the overlap of KEGG IDs within diatom (orange) and dinoflagellate (black) expression modules. Horizontal bars (left) indicate the number of KEGG IDs within a module (based on WGCNA), and vertical bars indicate the number of KEGG IDs within the set indicated by the dots and connecting lines in the lower panel: single dots indicate single modules, and multiple connected dots indicate overlapping sets.

Further investigation of module eigengenes revealed that 2 diatom modules (10 and 5) were highly expressed during periods of increases in chlorophyll and high relative abundance of rRNA transcripts (Day1 and Day 3) and were strongly correlated with phosphate and nitrite + nitrate concentrations (*p* <0.01; Supplementary Figure S6B). Only module 46, a dinoflagellate module, was strongly correlated with nutrient concentrations (*p* <0.01; Supplementary Figure S7B). Functional enrichment analysis for both algal groups was mostly inconclusive due to lack of annotations, a shortcoming extremely pronounced in dinoflagellates. In diatoms however, whereas enrichments in module 1 contained mostly biosynthesis processes, proteolysis enrichments were a consistent feature of the modules that correlated with advanced bloom stages (Supplementary Figures S6A, S8). Additionally, enrichments associated with human diseases were most enriched in modules 1 and 31, which correlated with peak bloom and decline during 2018 (Supplementary Figures S6A, S8). There was marginal overlap between diatom and dinoflagellate modules, and the largest overlapping set of KEGG IDs was between modules that were highly expressed during bloom dominance (Figure 5).

## 4. Discussion

The daily dynamics of the protistan community during two contrasting algal blooms were recently characterized via 18S rRNA gene transcript sequencing (Ollison et al., 2022). This study expands on the community succession patterns captured in Ollison et al. (2022) by examining expression patterns of KEGG annotated transcripts associated with changes in the relative abundances of numerically dominant diatom (2018) and dinoflagellate (2019) taxa during key phases of their respective blooms. We acknowledge that succession patterns due to movement of water masses are difficult to isolate, and may contribute to differences in community dynamics and gene expression patterns. However, the KEGG framework enabled broad comparisons between these phylogenetically distant algal guilds.

Diatoms *Thalassiosira* and *Pseud-nitzschia* were found to be the numerically dominant diatom taxa during 2018, while *Margalefidinium* and *Akashiwo* were the numerically dominant dinoflagellate taxa during 2019. Whereas *Thalassiosira* and *Pseudo-nitzschia* belong to phylogenetically distinct families, *Margalefidinium* and *Akashiwo* are both gymnodiniacean dinoflagellates. Analysis of gymnodiniacean dinoflagellate gene expression at the genus level was limited by the availability of dinoflagellate annotations, especially for *Margalefidinium*.

### 4.1. Physiologies underpinning diatom and dinoflagellate assemblages were distinct

Diatoms and dinoflagellates exhibited distinct transcriptional makeups regardless of year as exhibited by non-metric multi-dimensional scaling (Bray-Curtis) of gene expression profiles of *Thalassiosira*, *Pseudo-nitzschia*, and gymnodiniacean dinoflagellates (combined *Akashiwo* and *Margalefidinium* transcripts) during both 2018 and 2019 blooms. This NMDS resulted in three groupings where the primary grouping (NMDS1) was according to guild (diatom vs. dinoflagellate), a finding consistent with results of other recent comparative studies (Figure 2) (Alexander et al., 2015b; Harke et al., 2017; Zhang et al., 2019; Metegnier et al., 2020). For example, Harke et al. (2017) examined the transcriptional responses of species from phylogenetically distinct algal guilds to nutrient stress and found little variation between the responses of two distantly related mixotrophic dinoflagellate species, but in contrast found distinct physiological responses of these dinoflagellates compared to diatoms and haptophytes. Additionally, during the current study, WGCNA of diatom and dinoflagellate gene expression (i.e., *Thalassiosira* and *Pseudo-nitzschia* gene expression during 2018, and gymnodiniacean dinoflagellate gene expression during 2019) produced clusters of genes with similar expression dynamics (modules) that corresponded with each aforementioned growth phase during their respective bloom. Few KEGG IDs were shared between diatom and dinoflagellate modules despite their association with analogous growth phases, which underscores their physiological distinction (Figure 5).

### 4.2. Shifting physiological priorities of diatoms during bloom succession

The majority of diatom gene expression was dedicated to carbohydrate and fatty acid metabolism, especially during the 2018 bloom event when they were numerically dominant (Figures 3A,B), a finding that has been previously reported (Alexander et al., 2015a; Zhang et al., 2019). Additionally, energy/carbon metabolism categories that were differentially expressed by diatoms (here, *Thalassiosira* and *Pseudo-nitzschia*) were generally under-expressed during 2018. Most over-expression of categories within this grouping during 2018 was observed on Day 5 (max chlorophyll), the period of maximum relative abundances and chlorophyll concentrations (Figure 4A). All energy/carbon metabolism categories were subsequently under-expressed at the time of bloom decline (Day 9) with the exception of fatty acid metabolism, which were over-expressed throughout most of 2018. Genes associated with glycolysis and the TCA cycle were both over-expressed on Day 11.

Most nutrient processing categories that exhibited differential expression in diatoms during 2018 were over-expressed (Figure 4A). Few categories were differentially expressed on Day 3, which was a day that coincided with increased relative abundance of diatoms during 2018, but all categories were differentially expressed on Day 5. Notably all genes associated with nitrogen assimilation (GS/GOGAT, NR, Sulfite Oxidase, and other genes involved in assimilatory Nitrate reduction) were over-expressed during that period, and genes associated with phosphate, organic nitrogen (urea cycle), and extracellular ammonium uptake were under-expressed (Figure 4A). Similar over-expression of genes involved in extracellular nutrient uptake have been reported in previous studies examining nutrient limited diatoms in culture (Bender et al., 2014; Harke et al., 2017). Over-expression of genes associated with nutrient uptake during a period of low recorded nitrate + nitrite during this study may indicate the onset of nutrient deficient conditions on Day 5. We speculate that the under-expression of genes associated with nutrient uptake on Day 3, and the enrichment of genes associated with photosynthesis (Terpenoid backbone biosynthesis), cofactor and nucleic acid biosynthesis, and protein processing in modules 10 and 5, which were modules associated with diatom gene expression clusters on Day 1 and Day 3, may signify growth under replete conditions (Supplementary Figures S6A, S8). Fewer categories associated with nitrogen assimilation were differentially expressed by Day 11, but all categories associated with ammonium and nitrate uptake (AMT, NRT) as well as with the degradation of chitin were over-expressed. Decrease in genes associated with nitrogen assimilation but the continued over-expression of genes associated with nitrogen uptake may indicate depleted intracellular nitrogen and the onset of nitrogen limitation during this advanced bloom stage.

Most of the seven categories associated with other cellular processes were under-expressed. However, perhaps unsurprisingly, the nucleotide and amino acid metabolism category was over-expressed during the increase of diatom relative abundances (Day 3), and subsequently under-expressed during the periods of maximum abundance and decline.

Over-expression of genes associated with nucleotide and amino acid biosynthesis during a period of increasing relative abundances is consistent with diatom cell division and proliferation following nutrient upwelling as noted by Ollison et al. (Figure 1A of Ollison et al., 2022). Consistent with dissolved inorganic nitrogen drawdown and subsequent assimilation, all categories associated with nitrate and ammonium assimilation were subsequently over-expressed, and those associated with phosphate and organic nitrogen (urea cycle) were under-expressed on Day 5 (Figure 4A).

On Day 5, there was also a general over-expression of energy/carbon metabolism genes in diatoms, including genes involved in photosynthesis, the Calvin cycle, glycolysis, and fatty acid metabolism. The increase in organic carbon production at the height of the bloom presumably reflects the uptake and utilization of new, upwelled nitrogen for primary production, and the necessity of organic carbon skeletons for nitrogen assimilation (Hockin et al., 2012). All energy/carbon metabolism categories were under-expressed following depleted inorganic nitrogen concentrations (Ollison et al., 2022), and the number of differentially expressed categories associated with nitrogen assimilation decreased, and categories associated with ammonium and nitrate uptake were over-expressed. Differential expression patterns of both nutrient processing and energy/carbon metabolism categories during 2018 for diatoms mirrored proportional changes during 2019, where the overall proportion of energy/carbon metabolism categories were depressed and ammonium transporter was elevated during diatom decline under nutrient depleted conditions observed during the advanced stages of 2019 (Figure 3B, Supplementary Figures S1, S3). Studies examining the transcriptional response of diatoms to nitrogen limitation in culture have reported similar under-expression of genes associated with photosynthesis and central carbohydrate metabolism (Bender et al., 2014; Harke et al., 2017).

Nitrogen is an essential nutrient that is required for the biosynthesis of chlorophyll. Down-regulation of photosynthesis under nitrogen limitation is universal across distantly related photosynthetic organisms as chlorophyll is a nitrogenous compound, and decreasing its synthesis might lower nitrogen demand. Therefore, the under-expression of transcripts associated with photosynthesis and the Calvin cycle in this study is not surprising, and is in agreement with other studies (Mock et al., 2008; Hockin et al., 2012; Schmollinger et al., 2014). Likewise, upregulation of genes associated with nutrient uptake and catabolism of nitrogenous compounds such as chitin and amino acids has been reported under nutrient limitation across an array of phylogenetically distinct phytoplankton (Frischkorn et al., 2014; Liu et al., 2015; Cooper et al., 2016; Alexander et al., 2020).

Genes associated with the degradation of chitin were over-expressed in diatoms during Day 11, a period of decline under depleted inorganic nitrogen (Figure 4A). The proportion of chitinase transcripts were also greatly elevated during the advanced, low inorganic nitrogen stages of 2019 (Supplementary Figures S2B, S3B). Chitin is a nitrogenous compound produced by some diatoms as a means of controlling buoyancy by modulating the length of chitin spicules through the activity of chitinase (Durkin et al., 2009). *Thalassiosira* cells produce chitin fibers that extend from the theca through specialized pores (Blackwell et al., 1967; Herth and Schnepf, 1982), and chitinase has been identified in the genome of *T. pseudonana*; however, chitinase has not be identified in *Pseudo-nitzschia* (Armbrust, 2004; Di Dato et al., 2015). Chitin degradation may produce intracellular nitrogen that can be reallocated during periods of deficiency. Accordingly, the expression of chitinase by *Thalassiosira* during periods of nitrogen deficiency during both years of this study is consistent with recycled nitrogen from chitin degradation as an alternative means of nutrient acquisition for sustained growth under deficient extracellular inorganic nitrogen. Interestingly, genes putatively associated with endocytosis were over-expressed with each instance of chitinase over-expression. Diatoms are believed to be purely phototrophic, although they evolved from phagotrophic origins (Falkowski et al., 2004; Armbrust, 2009). The genes putatively associated with endocytosis may be deeply conserved and have been coopted for different purposes in the phagotrophic relatives of diatoms while serving in non-phagotrophic, catabolic pathways for assimilating intracellular nutrients in diatoms.

### 4.3. Diatoms are physiologically divergent under stress

*Thalassiosira* and *Pseudo-nitzschia* were the numerically dominant diatom taxa following upwelling during 2018. Ordination of their gene expression profiles grouped all *Thalassiosira* expression profiles (both 2018 and 2019) with *Pseudo-nitzschia* expression profiles observed during 2018 (Figure 2); however, *Pseudo-nitzschia* gene expression observed during 2019 formed an independent grouping (Figure 2). The proportion of functional categories within gene expression profiles of each diatom taxa illustrated that their profiles were mostly indistinguishable during 2018; however, their profiles were divergent during the later portion of the algal bloom in 2019 (Supplementary Figure S3). The most salient differences were chitinase expression by *Thalassiosira*, and the spike in the proportion of transcripts associated with the genetic information processing category by *Pseudo-nitzschia* during advanced stages of 2019, most of which coded for ribosomal proteins and tRNA synthetases. As noted in the previous section, *Thalassiosira* is a known producer of chitin and contains genes for the synthesis of chitinase within its genome (Armbrust, 2004; Durkin et al., 2009). We speculate that this species persisted more effectively than *Pseudo-nitzschia* under deficient inorganic nitrogen during advanced stages of 2019 through the use of recycled nitrogen from chitin degradation.

*Thalassiosira* and *Pseudo-nitzschia* evolved from phylogenetically distinct families whose last common ancestor may have diverged nearly 200 MYA (Falkowski et al., 2004; Strassert et al., 2021). The grouping of their expression profiles during 2018 and their dissimilarity during 2019 indicate that these two deeply diverged diatom taxa exhibited similar physiology under the upwelling conditions observed during 2018, while their physiologies diverged under more pronounced nutrient depletion observed during 2019. Studies contrasting genomes and transcriptomes among phylogenetically distinct diatoms have illustrated conserved traits that likely underpins their ability to thrive under high-nutrient settings such as costal upwelling regimes (Armbrust, 2004; Bowler et al., 2008; Bender et al., 2014; Di Dato et al., 2015; Alexander et al., 2015a). For example, Bender et al. (2014) found similarities in the regulation of carbohydrate and nutrient utilization among three phylogenetically distant diatoms—*Thalassiosira*, *Pseudo-nitzschia*, and *Fragilariopsis*—that may indicate pathway-level similarities governing diatom responses to environmental cues despite evolved differences in morphology and physicochemical requirements (Bender et al., 2014). However, chitinase expression by *Thalassiosira* during nutrient deficiency was one distinguishing feature identified in this study through contrasting diatom gene expression patterns (Supplementary Figure S3). Our results reveal that while the physiological strategies of *Thalassiosira* and *Pseudo-nitzschia* may be similar under favorable growth conditions, subtle differences in their physiologies may result in differential success under nutrient stress.

### 4.4. Shifting physiological priorities of dinoflagellates during their bloom

Ollison et al. (2022) reported stronger upwelling conditions during the diatom bloom in 2018, and prolonged nitrogen depletion during the dinoflagellate bloom in 2019. Gymnodiniacean dinoflagellates *Akashiwo* and *Margalefidinium* were minor components of the protistan community during 2018, but during 2019 they bloomed to high cell abundances and the maximum recorded chlorophyll concentration during their bloom was 60 μg/L chlorophyll, a concentration approximately six times the major bloom threshold for Santa Monica Bay (Seubert et al., 2013).

Surprisingly, comparatively few dinoflagellate transcripts exhibited differential expression (225 dinoflagellate vs. 1,138 diatom DEGs), and the majority were over-expressed relative to Day 1 during 2019 (Figure 4B). The exceptions were genes involved in glycolysis and nitrate uptake (NRT), both of which maintained under-expression throughout advanced bloom stages. The observed low levels of transcriptional activity in dinoflagellates has been previously reported in other studies that have examined the transcriptional responses of dinoflagellates to environmental cues (Alexander et al., 2015b; Murray et al., 2016; Harke et al., 2017; Cohen et al., 2021). Dinoflagellates exhibited increases in relative abundances coincident with increases in chlorophyll concentrations on Day 4. The observed over-expression of genes associated with photosynthesis, nucleotide and amino acid biosynthesis, and several ribosomal proteins on this day is expected under conditions of population growth (Figures 1B, 4B).

Genes associated with inorganic nitrate uptake were a relatively minor component of dinoflagellate profiles during 2019, and were under-expressed throughout advanced bloom stages; however, genes associated with ammonium assimilation (GS/GOGAT) and the urea cycle (arginase) were over-expressed throughout 2019 (Figures 3D, 4B). Arginine, an amino acid containing a nitrogenous side chain, is produced in the urea cycle as a precursor to ornithine and urea, and therefore may represent a possible nitrogen storage molecule (Allen et al., 2011). Over-expression of arginase may represent the catabolic breakdown of arginine and subsequent assimilation of organic ammonium (Hockin et al., 2012). The lack of gene expression associated with dissolved inorganic nitrogen uptake during 2019 may further indicate a preference for organic nitrogen sources during the 2019 bloom.

We speculate, based on their transcriptional patterns, that dinoflagellates during the 2019 bloom may have acquired significant nutrients for growth via heterotrophy (i.e., mixotrophically). Mixotrophy, which is the combination of heterotrophy and phototrophy, has been reported in *Akashiwo* and *Margalefidinium* (Nielsen and Kiørboe, 2015; López-Cortés et al., 2019; Yang et al., 2020). *Margalefidinium* in particular is thought to reach high abundances in natural ecosystems only when dissolved nutrients are supplemented with phagotrophy (Hofmann et al., 2021). The elevated proportion of transcripts putatively associated with phagotrophic consumption in dinoflagellate expression profiles in addition to their over-expression during their bloom under depleted inorganic nitrogen is consistent with phagotrophy as a means of acquiring organic carbon and nitrogen (Figure 4; Supplementary Figure S1). Fatty acid accumulation has been observed in nitrogen starved alga in culture (Li-Beisson et al., 2019). Although genes associated with fatty acid metabolism were expressed by dinoflagellates in this study during 2019, not 2018, these transcripts did not exhibit differential over- or under- expression. It is not clear if fatty acids were accumulated in dinoflagellate cells during 2019. It is possible that expression of genes associated with fatty acid metabolism were utilized in the catabolic breakdown of lipid membranes during prey consumption.

The same categories associated with heterotrophy and the utilization of organic nitrogen were less prominent in dinoflagellate expression profiles during 2018, and the elevated proportion of dinoflagellate transcripts associated with dissolved inorganic nitrogen uptake (NRT) and assimilation (NR) during upwelling conditions of 2018 are consistent with greater reliance on dissolved inorganic nitrogen during that year (Figures 3C vs. D; Supplementary Figures S2C,D). Diatoms typically out compete dinoflagellates under high-nutrient upwelling conditions due to their faster intrinsic growth rates, consistent with our observation of diatom dominance following the strong upwelling in 2018 (Margalef, 1978).

Nonetheless, it is unclear why these mixotrophic dinoflagellates appeared to prioritize dissolved inorganic nitrogen during this year, based on their transcriptional profiles, whereas organic and heterotrophically acquired nitrogen was prioritized during 2019. One possibility is the lack of suitable prey during 2018 relative to 2019. Ollison et al. (2022) reported that parasites were particularly pronounced during 2019 and increased in relative proportion coincident with the decline of non-dinoflagellate algae (Figures 3B of Ollison et al., 2022). They hypothesized that zoosporic cercozoan parasites may have facilitated the bloom of dinoflagellates either indirectly through the production of dissolved organic nutrients from diatom lysis, or directly as prey for phagotrophic consumption. Free-swimming zoosporic parasites are known to be grazed by microzooplankton and are thought to be an excellent food source in terms of shape, size and content (Kagami et al., 2007; Thieltges et al., 2013; Kagami et al., 2014). Although free-swimming parasites acting as prey for mixotrophic dinoflagellates has yet to be demonstrated, gene expression measured during this study is consistent with these dinoflagellates consuming prey during their bloom in 2019, perhaps due to higher abundances of parasites in that year but not 2018. More work examining these important agents is a necessary next step toward elucidating this possibility.

Categories associated with genetic information processing were over-expressed throughout all phases of the dinoflagellate bloom during 2019, and accounted for nearly half of dinoflagellate expression profiles (Figures 3C,D, 4B). The majority of transcripts in this category were genes that coded for snRNPs and ribosomal proteins, which are proteins involved in pre-mRNA splicing and translation, respectively, and is indicative of highly active splicing machinery coupled with protein translation in these species (Lerner et al., 1980). The majority of dinoflagellate genes are thought to be post-transcriptionally regulated with pre-mRNA transcripts processed through spliced leader trans-splicing (Zhang et al., 2009; Murray et al., 2016). Additionally, high numbers of introns per gene have been reported in dinoflagellates, and a recent study that reconstructed intron evolution in five dinoflagellate genomes found evidence for recently active Introners, which are a type of genetic element that creates copies of itself that insert into many genes across the genome (Roy et al., 2023). Furthermore, alternative splicing is a hallmark of eukaryotic post-transcriptional gene regulation in which alternatively spliced pre-mRNA transcripts (i.e., introns and exons) result in multiple distinct mRNA transcript isoforms with distinct, and sometimes antagonistic fates. Expressed isoforms may also be down-regulated through the splicing of poison exons (Gilbert, 1978; Lareau et al., 2007; Wang and Burge, 2008). It is not clear if the expression of alternatively spliced transcript isoforms in response to environmental cues, not over- or under-expression of the same isoform, may explain the low differential expression of genes in dinoflagellates here and across many studies (Alexander et al., 2015b; Murray et al., 2016; Harke et al., 2017; Cohen et al., 2021). The large genomes of dinoflagellates present formidable challenges in the study of their gene regulation. Accordingly, over 50% of dinoflagellate transcripts in this study lacked gene function or deeper taxonomic classification. However, sequencing of more non-model dinoflagellate genomes would improve our ability to investigate post-transcriptional gene regulation in this ecologically important guild.

## Data availability statement

The datasets presented in this study can be found in online repositories. The name of the repository and accession number can be found at: National Center for Biotchnology Information (NCBI) BioProject, https://www.ncbi.nlm.nih.gov/bioproject/, PRJNA1011812.

## Author contributions

GO: Conceptualization, Data curation, Formal analysis, Investigation, Methodology, Visualization, Writing – original draft, Writing – review & editing. SH: Methodology, Writing – review & editing. JH: Investigation, Methodology, Writing – review & editing. BS: Methodology, Resources, Writing – review & editing. JB: Methodology, Writing – review & editing. DC: Conceptualization, Funding acquisition, Project administration, Resources, Supervision, Writing – review & editing.
