# Supplementary material for: Physiology governing diatom vs. dinoflagellate bloom and decline in coastal Santa Monica Bay
Source: Front Microbiol. 2023 Nov 29;14:1287326. doi: 10.3389/fmicb.2023.1287326 (PMC10716250; doi:10.3389/fmicb.2023.1287326)
Supplement: Supplementary file 1 [file Data_Sheet_1.zip › DataSheet1/SuppFigures.docx]

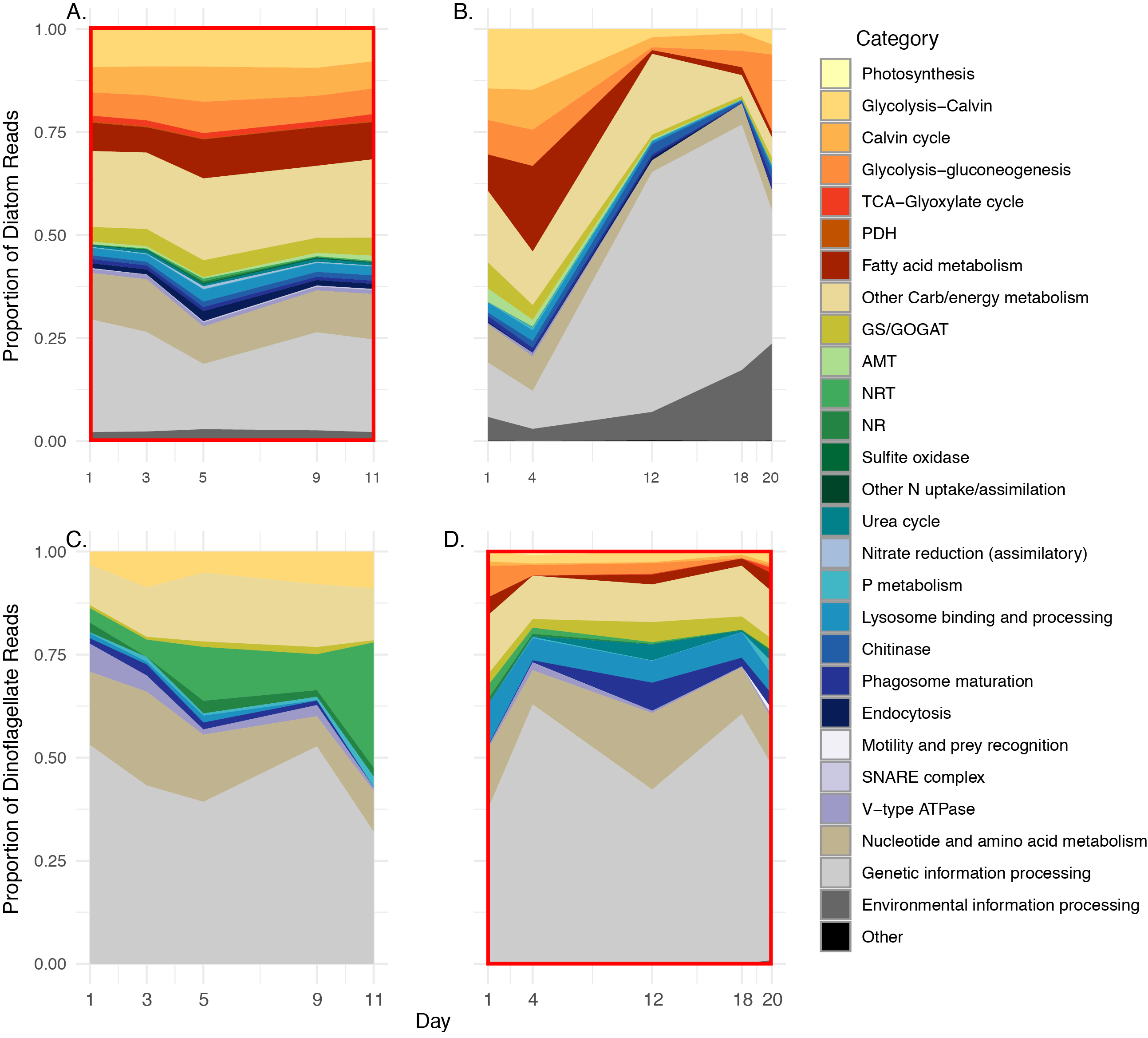


Figure S1


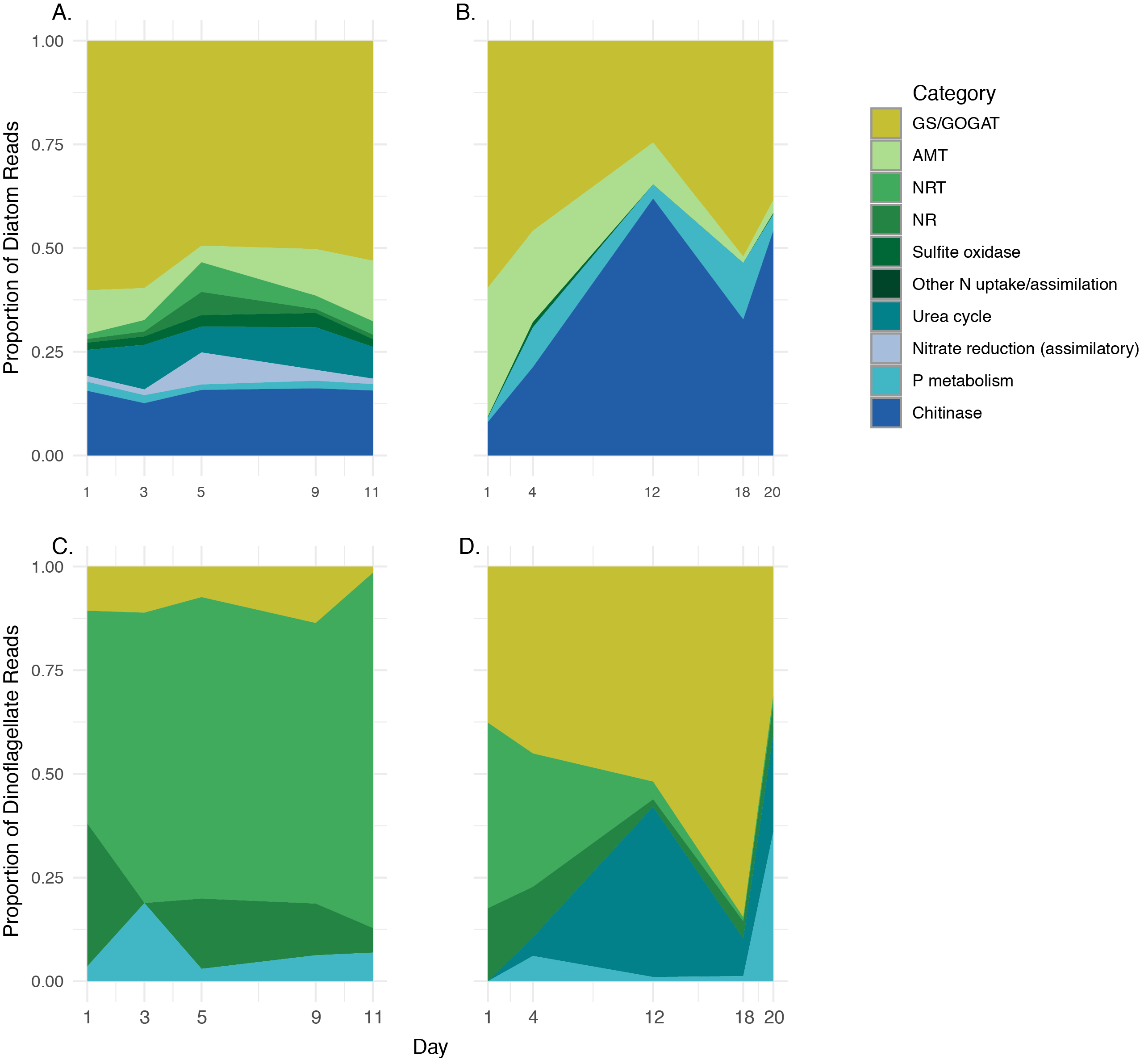


Figure S2


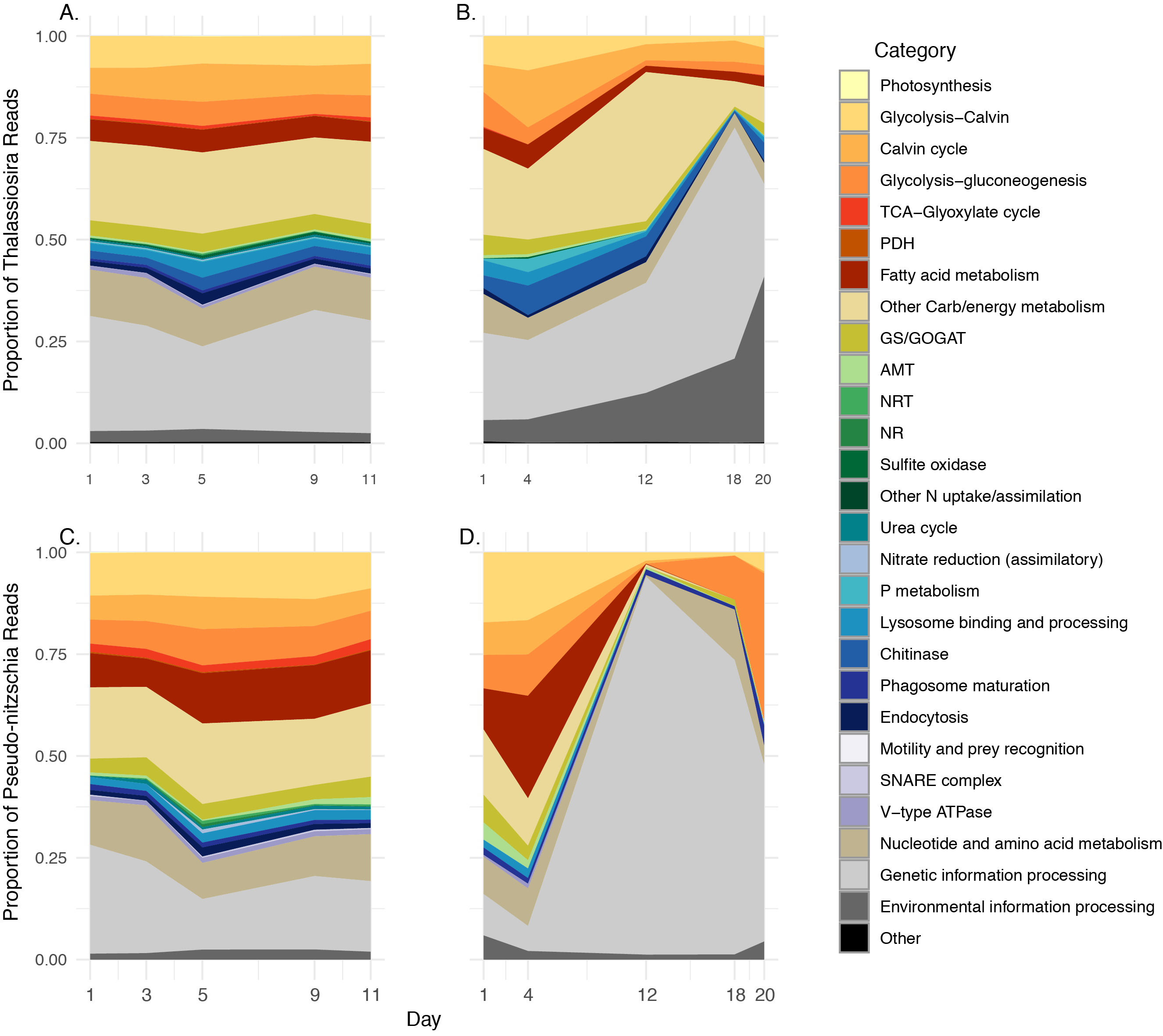


Figure S3


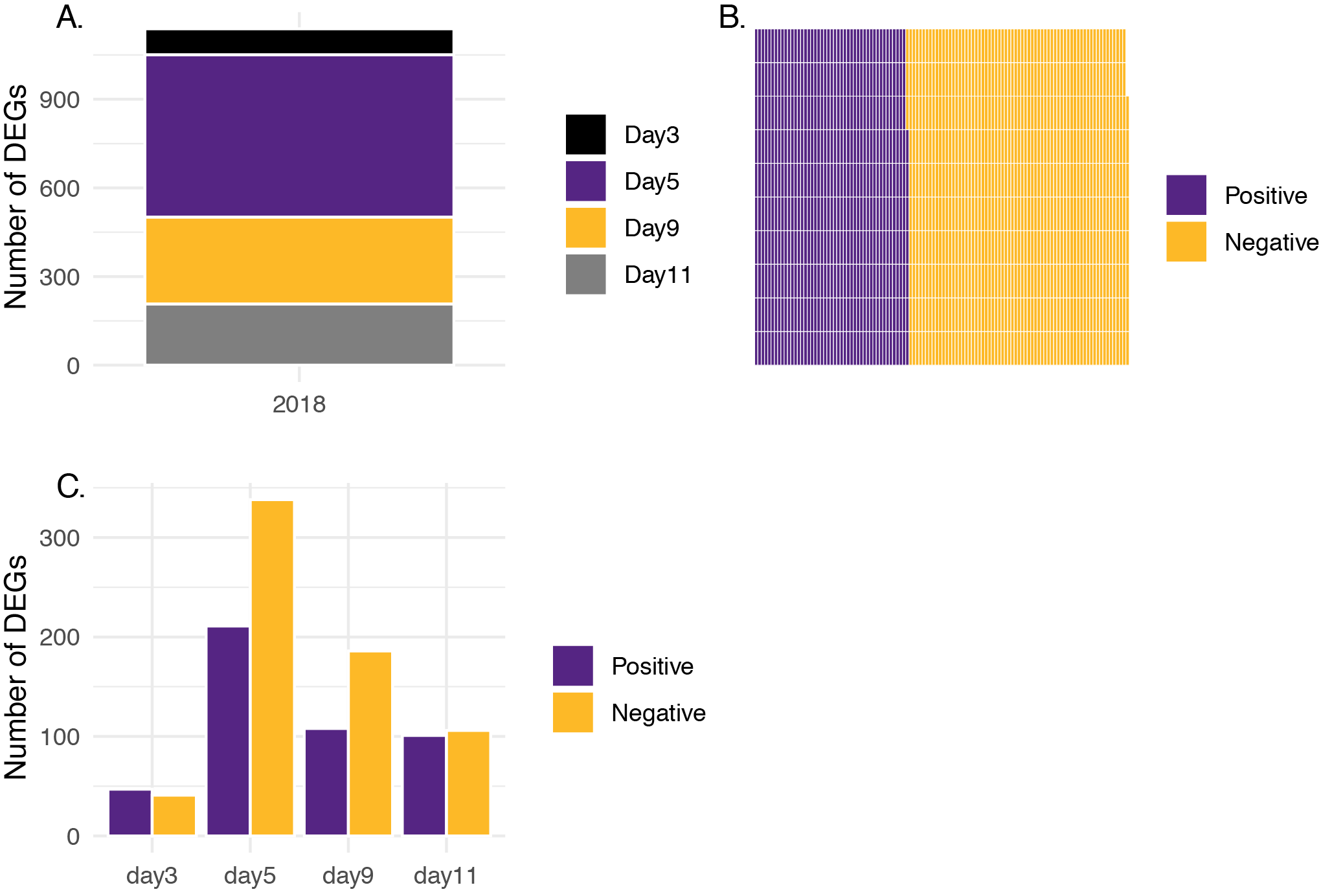


Figure S4


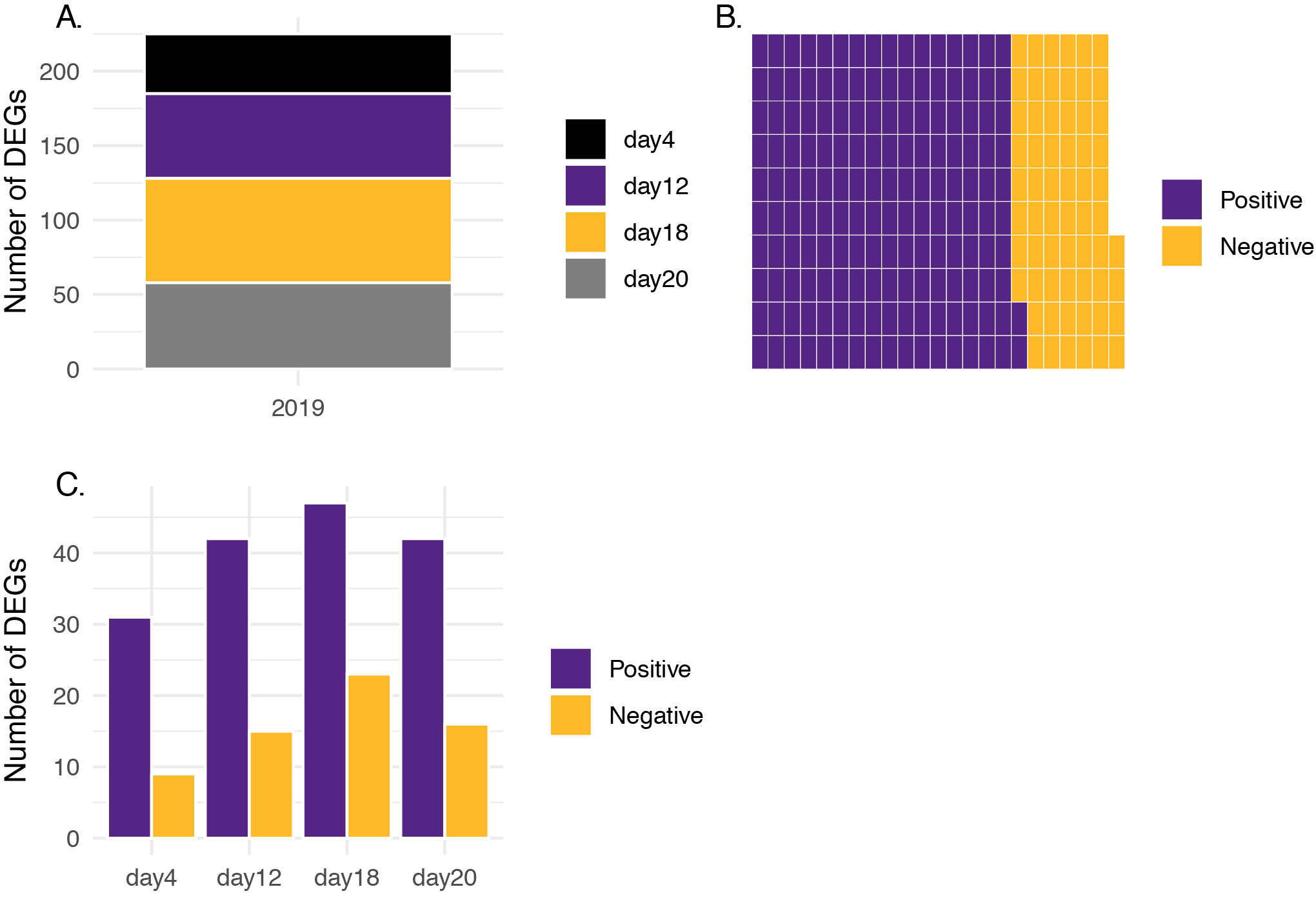


Figure S5


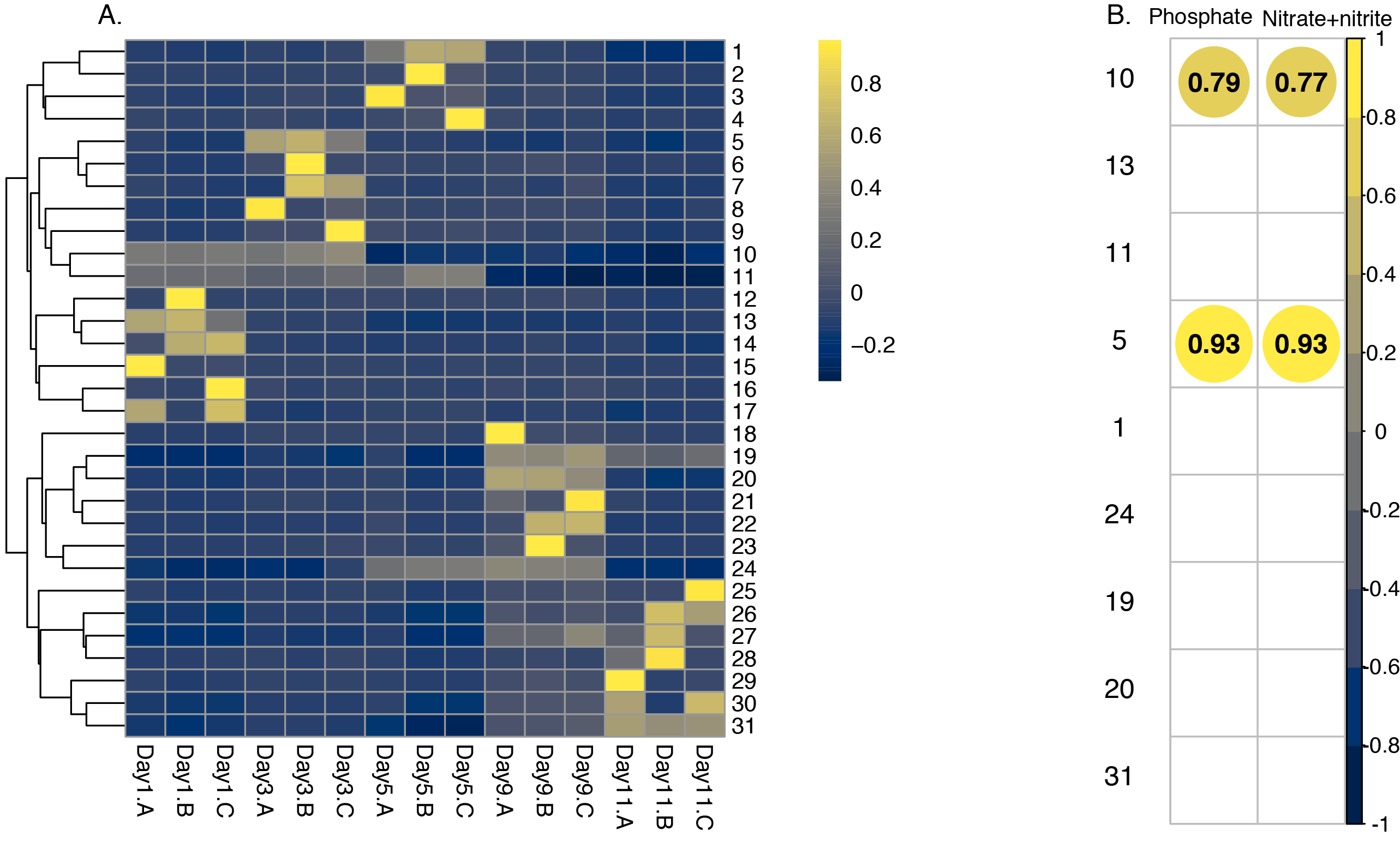


Figure S6


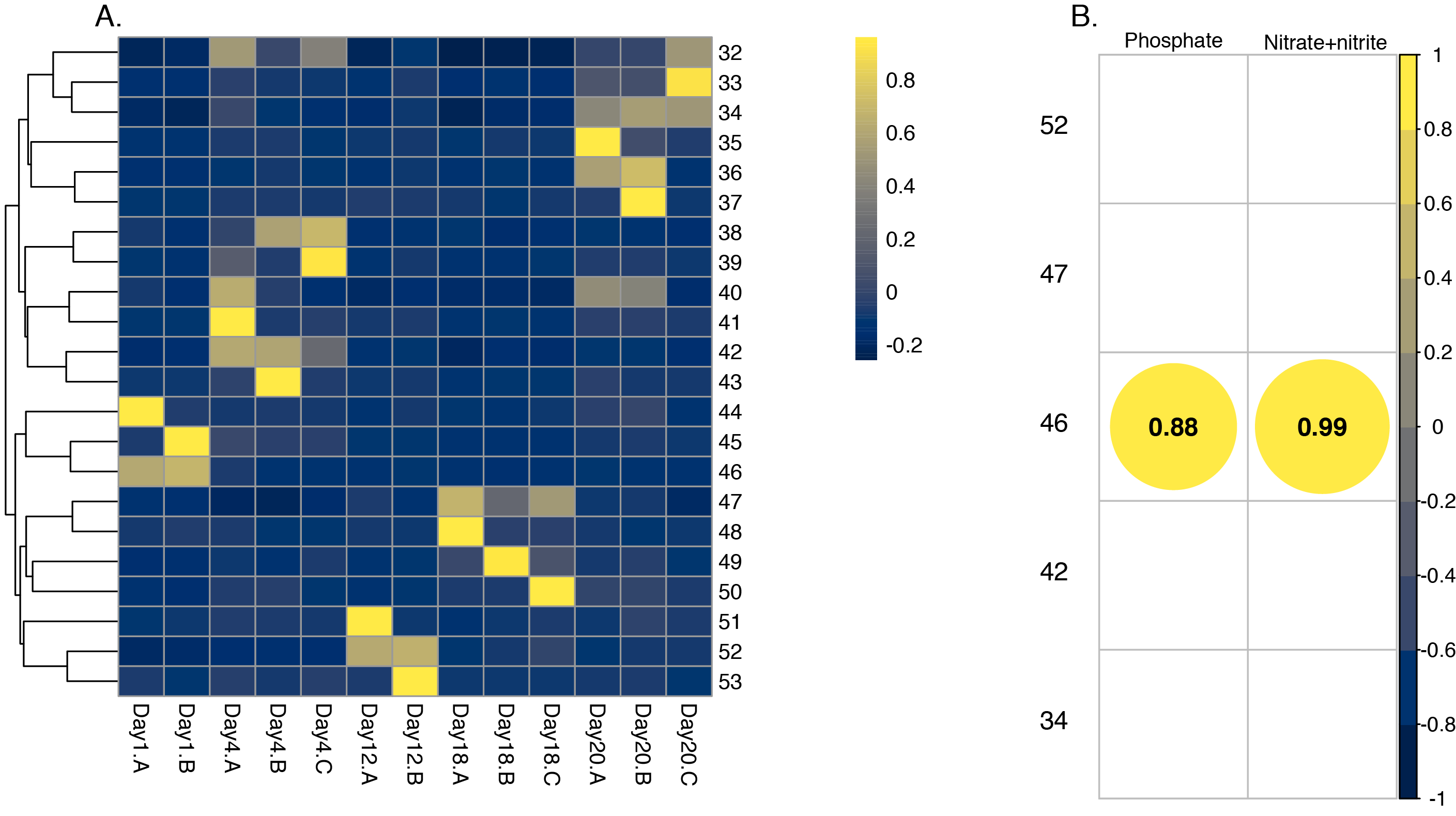


Figure S7


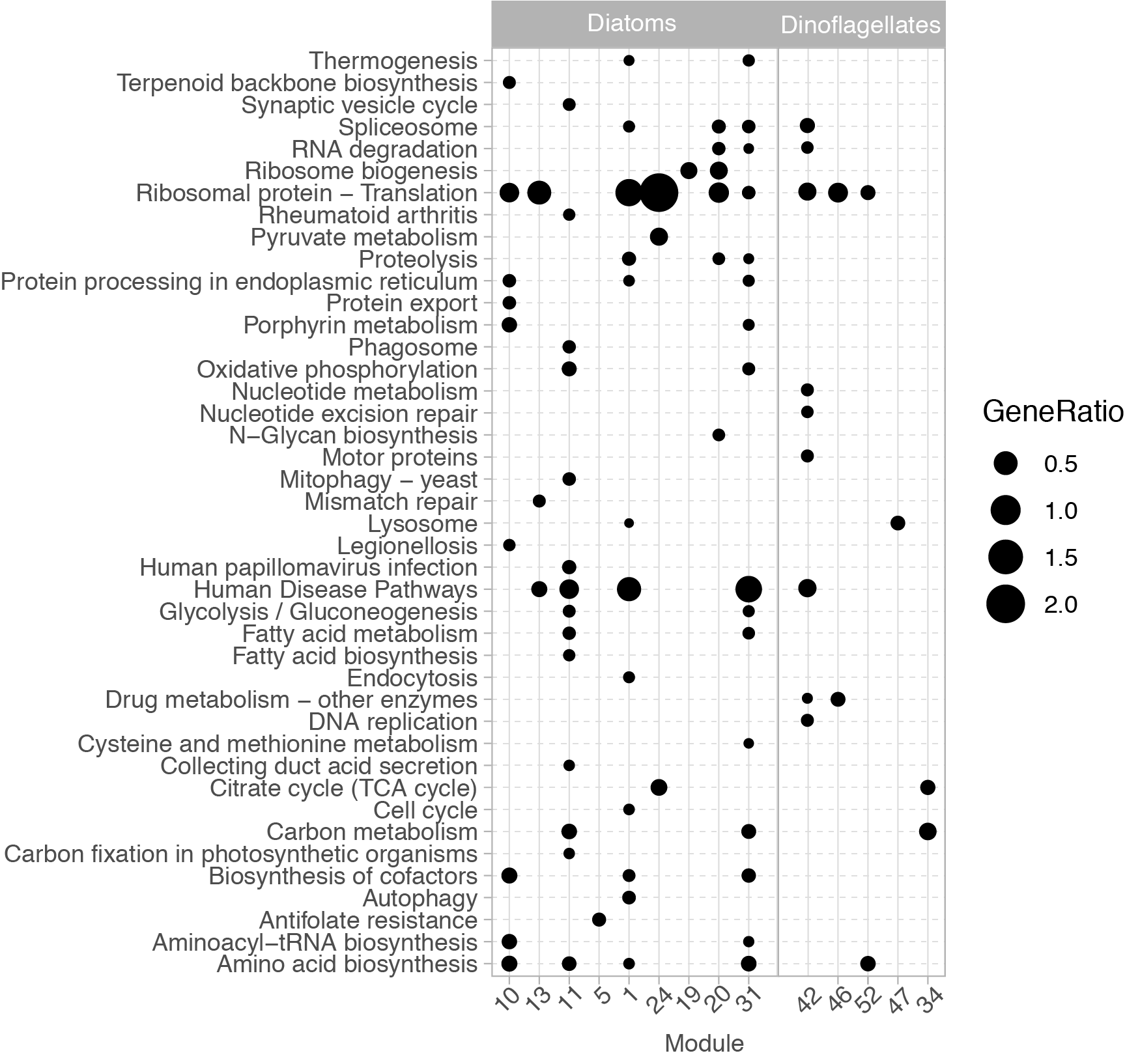


Figure S8

Figure S1: High resolution relative proportion of KEGG ID’d transcripts on five days during 2018 (A,C) and 2019 (B,D) grouped as categories that were expressed by diatoms (A,B) and dinoflagellates (C,D) during 2018 and 2019. Red boxes indicate years of numerical dominance of diatoms (A) and dinoflagellates (D).

Figure S2: Relative proportion of diatom (A,B) and dinoflagellate (C,D) functional categories associated with nutrient utilization.

Figure S3: High resolution relative proportion of KEGG ID’d transcripts on five days during 2018 (A,C) and 2019 (B,D) grouped as categories that were expressed by *Thalassiosira* (A,B) and *Pseudo-nitzschia* (C,D) during 2018 and 2019.

Figure S4: Number and proportion of differentially expressed diatom genes across sampling days (A), in addition to the fraction (B) and distribution (C) of differentially expressed genes with positive (purple) vs negative (yellow) log_2_ fold change values relative to Day 1 during 2018.

Figure S5: Number and proportion of differentially expressed dinoflagellate genes across sampling days (A), in addition to the fraction (B) and distribution (C) of differentially expressed genes with positive (purple) vs negative (yellow) log_2_ fold change values relative to Day 1 during 2019.

Figure S6: Module gene expression across all replicates collected on each sampling day (A) and correlations between module eigengenes and nutrient concentrations (B) during 2018. Only module eigengene – environment correlations with *p*-values < 0.01 are shown. Modules were constructed from *Thalassiosira* and *Pseudo-nitzschia* gene expression during 2018.

Figure S7: Module gene expression across all replicates collected on each sampling day (A) and correlations between module eigengenes and nutrient concentrations (B) during 2019. Only module eigengene – environment correlations with *p*-values < 0.01 are shown. Modules were constructed from gymnodiniacean dinoflagellate gene expression during 2019.

Figure S8: Functional terms that were significantly enriched in diatom modules (*Thalassiosira* and *Pseudo-nitzschia*) during 2018 (left panel) and gymnodiniacean dinoflagellate modules during 2019 (right panel; GeneRatio > 0.025 and *p*-value < 0.01).

10.0 Table legends

Table S1: Raw gene expression tables from 2018 and 2019 sampling periods, from which normalized tables containing filtered numerically dominant diatom and dinoflagellate taxa can be produced using R code provided.

Table S2: Diatom and dinoflagellate genes exhibiting significant differential expression with magnitude of log_2_ fold change (LogFC), KEGG Orthology ID (KO), and day examined relative to Day 1 (Contrast).
